# Synaptotagmin-4 induces anhedonic responses to chronic stress via BDNF signaling in the medial prefrontal cortex

**DOI:** 10.1038/s12276-024-01156-8

**Published:** 2024-02-01

**Authors:** Jeongseop Kim, Sihwan Seol, Tae-Eun Kim, Joonhee Lee, Ja Wook Koo, Hyo Jung Kang

**Affiliations:** 1 Emotion, Cognition & Behavior Research Group, Korea Brain Research Institute (KBRI), Dong-gu, Daegu, 41062 Republic of Korea; 2 Department of Brain Sciences, Daegu Gyeongbuk Institute of Science and Technology (DGIST), Dalseong-gun, Daegu, 42988 Republic of Korea; 3 Department of Life Science, Chung-Ang University, Seoul, 06974 Republic of Korea

**Keywords:** Stress and resilience, Depression, Behavioural genetics, Transcriptomics, Mouse

## Abstract

Stressful circumstances are significant contributors to mental illnesses such as major depressive disorder. Anhedonia, defined as loss of the ability to enjoy pleasure in pleasurable situations, including rewarding activities or social contexts, is considered a key symptom of depression. Although stress-induced depression is associated with anhedonia in humans and animals, the underlying molecular mechanisms of anhedonic responses remain poorly understood. In this study, we demonstrated that synaptotagmin-4 (SYT4), which is involved in the release of neurotransmitters and neurotrophic factors, is implicated in chronic stress-induced anhedonia. Employing chronic unpredictable stress (CUS), we evaluated two subpopulations of mice, susceptible (SUS, anhedonic) and resilient (RES, nonanhedonic), based on sucrose preference, which was strongly correlated with social reward. The FosTRAP (targeted recombination in active populations) system and optogenetic approach revealed that neural activity in the medial prefrontal cortex (mPFC) was significantly associated with CUS-induced anhedonic behavioral phenotypes. By conducting weighted gene coexpression network analysis of RNA sequencing data from the mPFC of SUS and RES mice, we identified *Syt4* as a hub gene in a gene network that was unique to anhedonia. We also confirmed that *Syt4* overexpression in the mPFC was pro-susceptible, while *Syt4* knockdown was pro-resilient; the pro-susceptible effects of SYT4 were mediated through a reduction in brain-derived neurotrophic factor (BDNF)-tropomyosin receptor kinase B (TrkB) signaling in the mPFC. These findings suggest that SYT4-BDNF interactions in the mPFC represent a crucial regulatory mechanism of anhedonic susceptibility to chronic stress.

## Chronic stress and depression: synaptotagmin-4 as a key player

Depression is a complex mental health disorder that can significantly impact a person’s life. One symptom, anhedonia, is particularly difficult to treat. Researchers have been exploring why some people develop anhedonia and others don’t, especially under stress. The study, led by Jeongseop Kim and Sihwan Seol, aimed to discover the biological processes behind anhedonia. Using mice, the team exposed them to chronic unpredictable stress and observed their behaviors. They found that not all mice responded to stress with depressive-like behaviors, indicating individual differences in stress resilience. The researchers concluded that SYT4 and BDNF play crucial roles in the development of anhedonia and stress-induced depression. This study advances our understanding of the molecular basis of depression and could lead to new treatments targeting these mechanisms. This summary was initially drafted using artificial intelligence, then revised and fact-checked by the author.

## Introduction

Depression is a common and severe mental illness that involves a persistent feeling of sadness and forfeiting pleasure or motivation^1^. Anhedonia, an impaired ability to experience pleasure and learn about rewards, has been used clinically as a criterion for the diagnosis of depression^2^. In a previous clinical study, anhedonia, along with a depressed mood, was found to outrank other depressive symptoms, including suicidal ideation, as the most fundamental symptom for the diagnosis of depression^2^. Anhedonia can be broadly divided into two categories: physical anhedonia, characterized by a lack of tactile pleasure, such as pleasure in food and sex, and social anhedonia, characterized by increased apathy in interactions and a lack of pleasure in social situations^3,4^. Notably, anhedonia does not typically respond to initial depression treatments^5,6^ and is usually the last symptom to resolve in clinical depression^7^. It is also a strong predictor of suicidal potential^8^ and treatment-resistant depression^5^, and stress-induced depression has been clearly related to anhedonia in humans and rodents. It is generally recognized that the frontal lobe-oriented neural network is particularly important in anhedonia;^9,10^ however, the underlying neurobiological mechanisms of anhedonia remain unclear.

In humans, a chronically stressful life is directly correlated with depressive disorders^11,12^. Several animal models, such as the model generated by chronic unpredictable stress (CUS), which is one of the most reliable and widely used stress paradigms, have been developed to replicate this phenomenon in animals. CUS causes various depressive-like phenotypes, including anhedonic-like behaviors^13,14^. Interestingly, not all stress-exposed individuals exhibit depressive-like behaviors in response to chronic stress. Some stressed animals maintain normal psychological functioning, suggesting that interindividual differences in reward responsiveness occur after chronic stress^15,16^.

Recent transcriptomic studies have identified broad transcriptional changes across brain regions, including the medial prefrontal cortex (mPFC), in stress-resilient and stress-susceptible mice^17–19^. This suggests that stress resilience is an active homeostatic response to stress rather than a lack of stress susceptibility^19^ and that individual differences in depressive-like behaviors such as anhedonia should be considered in physiological and molecular studies^17–19^. Network-based analysis methods, such as weighted gene coexpression network analysis (WGCNA), which defines gene networks from RNA sequencing data by clustering genes into modules according to coordinated transcriptional alterations, are helpful for studying the functional gene networks underlying individual differences^18^. WGCNA can identify “stress susceptibility networks” that rely on the expression of key module hub genes, which affect the expression of other genes within modules and overall stress susceptibility when their expression is manipulated in vivo^17,20,21^.

In the present study, we discovered that *synaptotagmin-4* (*Syt4)*, an immediate-early gene^22^ that modulates the interaction of neurotransmitter release, synaptic transmission, and Ca^2+^ entry^23,24^, governs a transcriptionally active gene network unique to anhedonia in the mPFC. SYT4 is involved in the negative regulation of brain-derived neurotrophic factor (BDNF) release, particularly in an activity-dependent manner^25,26^. Reduced levels of BDNF are known to be associated with the manifestation of depressive symptoms in both humans and animals^27–30^. In contrast, activity-dependent release of BDNF in the mPFC plays a vital role in mediating rapid antidepressant effects^31–33^. Our data demonstrated that SYT4 in the mPFC mediates CUS-induced anhedonic behaviors through a reduction in BDNF–TrkB signaling. Taken together, our findings suggest that the SYT4–BDNF interplay within the mPFC plays critical roles in anhedonia by modulating the major transcriptional network involved in stress susceptibility.

## Materials and methods

A detailed description of the experimental methods, including behavioral experiments, stereotaxic surgery, optogenetics, sequencing, WGCNA, drug infusion, qRT‒PCR, western blotting, and fluorescence in situ hybridization (FISH), is provided in the Supplementary Materials and Methods.

## Results

### Chronic unpredictable stress (CUS) induces various depressive-like behaviors

We exposed mice to 4 weeks of CUS and measured depressive-like behaviors via several behavioral tests (Supplementary Table 1). During the first 3 days after the completion of CUS exposure, we conducted a sucrose preference test (SPT), sociability test (ST), and social novelty discrimination (SND) test to determine the effects of CUS on physical, social anhedonia, and social novelty recognition outcomes in mice (Supplementary Fig. 1a). Before undergoing CUS (pre-CUS), all the experimental groups preferred the sucrose reward. The body weight and sucrose preference of the CUS-exposed group were significantly lower than those of the stress-naïve control (CTRL) group (Supplementary Fig. 1b, d). We next investigated whether the preference for sucrose after CUS was influenced by the demand for sucrose prior to stress using one-way analysis of covariance (ANCOVA) but found no significant effect of CUS on the relationship between sucrose preference before and after CUS (Supplementary Fig. 1c). Next, we performed the ST and SND test to investigate social anhedonia and the perception of novel experiences, respectively. CUS mice showed a lower preference for the cage containing a stimulus mouse than for an empty cage (Supplementary Fig. 1e). Similarly, stressed mice were more inclined to seek out known mice and less likely to acknowledge enclosures containing novel mice (Supplementary Fig. 1f). Interestingly, there was no discernible relationship between the results of the ST and SND test (Supplementary Fig. 1g).

Immediately after the SND experiment, over the next day, we conducted novelty-suppressed feeding (NSF), elevated plus maze (EPM), and forced swim test (FST) experiments to determine the least to most stressful behaviors and to examine anxiety- and despair-like behaviors. The CUS mice exhibited considerably reduced feeding activity in the unfamiliar environment but ate the same amount of food in their home cages within the first 5 min after NSF testing (Supplementary Fig. 1h, i). Additionally, compared to the CTRL mice, the CUS mice spent less time in the open arms and more time in the closed arms of the EPM (Supplementary Fig. 1j). Moreover, during the FST, the CUS mice struggled less than the CTRL mice did (Supplementary Fig. 1k). These data suggest that CUS induces anxiety- and despair-like behaviors in mice, while 4 weeks of CUS significantly induces various depressive symptoms, including weight alterations, social novelty discrimination, and anhedonia, in both the physical and social domains.

### Anhedonic behavioral traits can be used to separate susceptible and resilient subphenotypes of mice with depressive-like behaviors

To determine the most appropriate behavioral models for examining individual variations in depressive behaviors following CUS, we next evaluated different aspects of depressive-like behaviors using the SPT, ST, FST, SND test, and EPM test and validated the correlations between each behavior. One-way ANCOVA was used to determine how strongly each of the five behavioral outcomes was correlated with each other. The results showed that individual variations in SPT, a representative anhedonia behavioral experiment, were strongly correlated with individual variations in all depression-like behavior tests, except for the EPM test, which assesses anxiety-related behavior (Supplementary Fig. 2a–e). Moreover, when the correlation of each depression-like behavior with the ST was determined, there was no correlation with any behavior except SPT. Similarly, as shown in Supplementary Fig. 1g, there was no discernible correlation between individual variations in the results of the ST and SND test, while the SND test, in contrast to the ST test, showed minimal connection to the FST. Intriguingly, the results of the EPM test were not significantly correlated with any of the depressive-like behaviors. The SPT results were strongly correlated with the ST, SND, and FST results, suggesting that the SPT may serve as a general hub indicator of depressive-like behaviors.

**Fig. 1:**
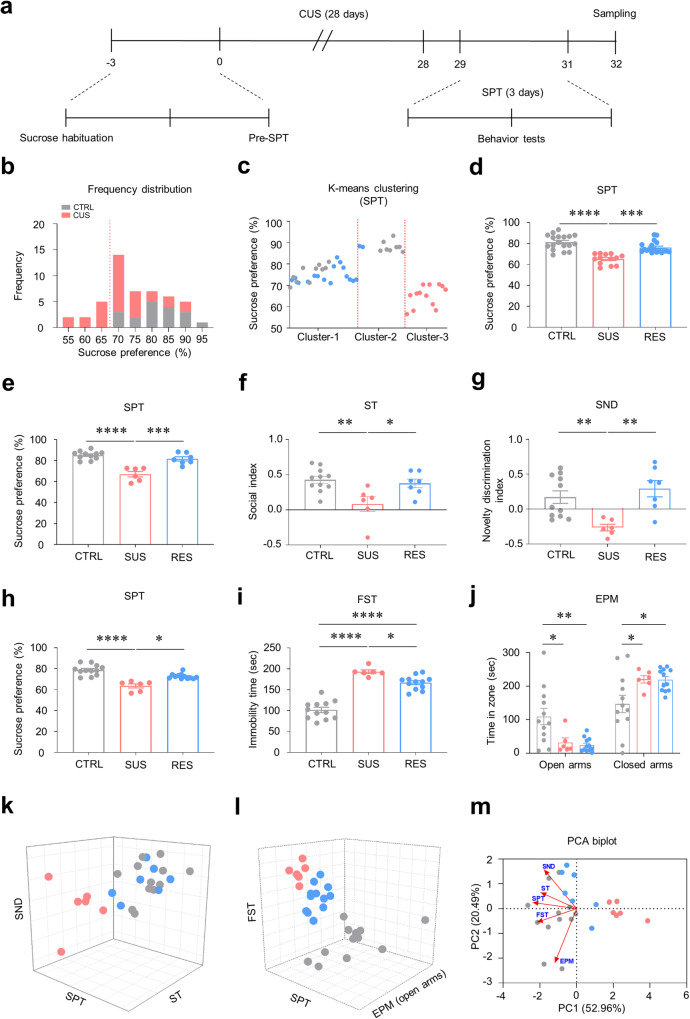
Classification of subpopulations according to chronic stress-induced behavioral phenotypes. **a** Experimental procedures for chronic unpredictable stress (CUS). **b** Frequency distribution histogram for the sucrose preference (*n* = 18, 31). **c** Separation of mouse populations by *K*-means clustering based on the sucrose preference test (SPT) dataset in Fig. 1b. **d** Susceptible (SUS, pink) mice showed a markedly reduced sucrose preference compared to resilient (RES, blue) and control (CTRL, gray) mice (Kruskal–Wallis test with Dunn’s test, *H* = 29.02, *p* < 0.0001; *n* = 18, 13, 18). **e** Sucrose preference test in mice subjected to a social behavior test (one-way ANOVA with Tukey’s test, *F*_2,21_ = 26.19, *p* < 0.0001; *n* = 11, 6, 7). **f** SUS mice exhibited social anhedonia, which was assessed via a sociability test (ST), while RES and CTRL mice did not (one-way ANOVA with Tukey’s test, *F*_2,21_ = 6.785, *p* = 0.0053, *n* = 11, 6, 7). **g** SUS mice showed impaired social cognition, as determined via a social novelty discrimination (SND) test, compared to RES and CTRL mice (one-way ANOVA with Welch’s test, *W*_2,12.41_ = 15.31, *p* = 0.0004, *n* = 11, 6, 7). **h** Sucrose preference test in mice subjected to anxiety- and depressive-like behavior (Kruskal–Wallis test with Dunn’s test, *H* = 19.53, *p* < 0.0001; *n* = 12, 6, 12). **i** Both SUS and RES mice exhibited increased immobility times in the forced swim test (FST) compared to those of the CTRL mice. Compared with RES mice, SUS mice exhibited modestly increased despair behavior (one-way ANOVA with Tukey’s test, *F*_2,27_ = 65.88, *p* < 0.0001, *n* = 12, 6, 12). **j** Both SUS and RES mice spe*n*t less time in the open arms but more time in the closed arms in the elevated plus maze (EPM) than did CTRL mice (two-way ANOVA with Tukey’s test: group effect, *F*_1,54_ = 73.69, *p* < 0.0001; maze effect, *F*_2,54_ = 0.0756, *p* = 0.9273; interaction, *F*_2,54_ = 12.02, *p* < 0.0001, *n* = 12, 6, 12). **k** Three-dimensional plot de*p*icting the results of the SPT, ST, and SND test for each of the SUS, RES, and CTRL mice in the same cohort. **l** Three-dimensional plot depicting the SPT, FST, and EPM test resu**l**ts for each of the SUS, RES, and CTRL mice in the same cohort. **m** Principal component analysis (PCA) biplot analysis of five representative variables (SPT, ST, SND, FST, and EPM) from the CUS subphenotypes. The first two principal components (PCs) explained 86.24% of the total variance, with PC1 explaining 52.96% and PC2 explaining 20.49%. The biplot shows clear partitioning of SUS mice from RES and CTRL mice along PC1. The variable vector, SPT, was the closest vector to the PC1 axis, followed by FST and ST. In contrast, the SND and EPM variable vectors were not closely plotted to either PC1 or PC2. The red arrows indicate the direction and length of the vectors of the variables. Each colored dot represents the sampling point for each subphenotype (*n* = 12, 6, 7). ^*^p < 0.05, ^**^p < 0.01, ^***^p < 0.001, ^****^p < 0.0001. The bar graphs show the mean ± SEM.

The frequency distribution histogram revealed that the CUS group had a broader distribution of sucrose preferences than the CTRL group (Fig. 1a, b). However, the sucrose preference of some CUS mice was similar to that of CTRL mice. To pinpoint the neurobiological mechanisms of individual differences after CUS, precise criteria are needed to separate subpopulations exhibiting distinct depressive-like behavioral phenotypes. To accomplish this, we employed *K*-means clustering, an unsupervised learning algorithm that divides groups according to similarities among the data. Through *K*-means clustering, we separated the data into three categories: a group that preferred a sucrose reward despite being exposed to CUS (RES, Cluster-1); a group that showed considerably reduced sucrose reward responsiveness due to CUS (SUS, Cluster-3); and a control group (CTRL), which was widely mapped to Cluster-2 and Cluster-1 (Fig. 1c, d). Further analysis and behavioral tests revealed that the subpopulations according to sucrose preference exhibited distinct behavioral phenotypes. Compared with RES and CTRL mice, SUS mice with a substantially reduced desire for sucrose exhibited severe social anhedonic behaviors in the ST and impaired social novelty recognition in the SND test (Fig. 1e–g). These findings are consistent with our results showing that individual variations in depressive behaviors in the SPT are strongly correlated with individual performance in the ST and SND test (Supplementary Fig. 2e).

We next conducted additional experiments to investigate how the CUS-induced individual variations in anhedonic behaviors observed in the SPT (Fig. 1h) are correlated with behavioral performance in the FST and EPM test. In the FST, the immobility time of the SUS mice was longer than that of the RES and CTRL mice. However, compared with the CTRL mice, the RES mice also exhibited increased behavioral despair (Fig. 1i). In the EPM test, both SUS and RES mice displayed higher levels of anxiety than CTRL animals (Fig. 1j). These data suggest that anxiety- and anhedonia-like behaviors are not connected in terms of individual variations, while the depressive-like subphenotypes observed in the SPT were most strongly associated with the ST and SND test (Fig. 1k, l). We next conducted principal component analysis (PCA), a multivariate analysis approach, employing data obtained from the SPT, ST, FST, SND test, and EPM test after CUS. The PCA biplot showed that the SPT was most strongly correlated with principal component 1 (PC1), followed by the ST and FST. In contrast, the SND and EPM results were not strongly correlated with PC1 or PC2. Additionally, the SPT, ST, and SND test variables were mapped onto the same dimensions, suggesting that all three variables are grouped into clusters that share common features or characteristics. The FST was located close to the SPT, suggesting a strong association between the two (Fig. 1m). Notably, the SUS group was distinct from the RES or CTRL groups in PC1, which indicates that the separation of these behavioral subphenotypes is based mainly on PC1. The SPT, the variable most strongly correlated with PC1, may be the most important factor explaining the overall grouping of the behavioral subphenotypes.

Finally, we assessed whether the SPT was a general hub indicator that classified individual variations in depressive behaviors by applying *K*-means clustering to individual behavioral data other than those obtained from the SPT (Supplementary Fig. 3). Clustering of the ST and SND data revealed that, compared with the ST-RES, SND-RES, and CTRL mice, both the ST-SUS and SND-SUS mice exhibited clear differences in social and novelty discrimination indices (Supplementary Fig. 3c, d, i, j). In the SPT, both ST- and SND-SUS mice exhibited a lower sucrose preference than the CTRL animals, whereas the ST- and SND-RES mice were statistically indistinguishable from the CTRL and SUS groups (Supplementary Fig. 3e, k). When clustered based on the ST data, the ST-RES mice showed a significant decrease in perceived social novelty compared to the CTRL mice (Supplementary Fig. 3f, g). In contrast, when the patients were clustered based on SND data, there was no significant difference in social anhedonia (Supplementary Fig. 3l, m). Similarly, when clustered based on the FST and EPM data, the FST- and EPM-RES mice did not significantly differ from the FST- and EPM-SUS mice in terms of behavioral performance in the SPT, FST, and EPM test (Supplementary Fig. 4). Collectively, these findings demonstrate that the SPT is the most appropriate test for evaluating individual variations in CUS-induced depressive-like behaviors.

### The mPFC showed the greatest association with anhedonic behaviors

**Fig. 2:**
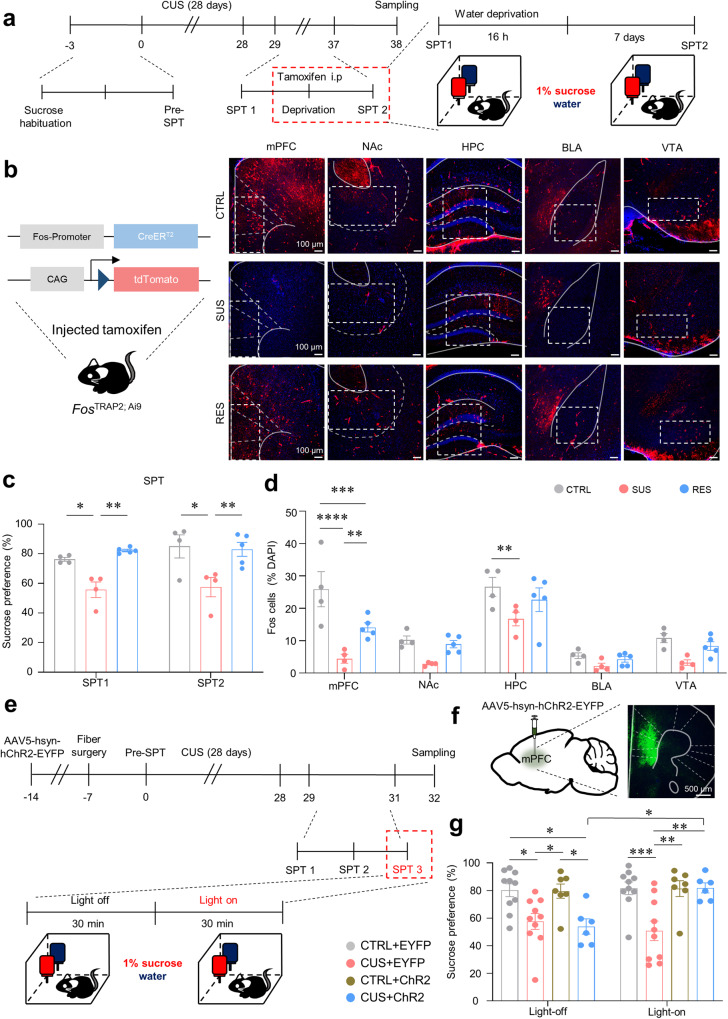
Chronic stress-induced anhedonia is mediated by neural activity in the mPFC. **a** Experimental procedures for capturing activated neurons during anhedonic behaviors. **b** Schematic of *Fos*^TRAP2; Ai9^ induction by tamoxifen (left) and representative images of c-Fos expression (red) in brain regions activated during the two-bottle sucrose preference test after CUS in CTRL, SUS, and RES mice (right, scale bars: 100 µm). **c** Sucrose preference before (SPT1) and after i.p. injection of tamoxifen (SPT2; two-way ANOVA with Tukey’s test: group effect, *F*_2,20_ = 17.19, *p* < 0.0001; time effect, *F*_1,20_ = 0.8945, *p* = 0.3555; interaction, *F*_2,20_ = 0.3897, *p* = 0.6823, *n* = 4, 4, 5). **d** c-Fos+ cell numbers in various brain regions in the limbic system, including the mPFC (two-way ANOVA with Tukey’s test: group effect, *F*_2,50_ = 25.11, *p* < 0.0001; region effect, *F*_4,50_ = 34.36, *p* < 0.0001; interaction, *F*_8,50_ = 2.535, *p* = 0.0212, *n* = 4, 4, 5). **e** Experimental procedure for photoactivation during the SPT after CUS. **f** A representative image of the channelrhodopsin-2 (ChR2) virus and optic fiber implantation in the PFC (scale bar: 500 µm). **g** Comparison of sucrose solution intake between groups before and after ChR2 stimulation after CUS (two-way ANOVA with Tukey’s test: group effect, *F*_3,58_ = 11.39, *p* < 0.0001; stimulation effect, *F*_1,58_ = 2.220, *p* = 0.1417; interaction, *F*_3,58_ = 3.002, *p* = 0.0377, *n* = 10, 10, 7, 6). ^*^p < 0.05, ^**^p < 0.01, ^***^p < 0.001. ^****^p < 0.0001. The bar graphs show the mean ± SEM. mPFC medial prefrontal cortex, NAc nucleus accumbens, HPC hippocampus, BLA basolateral amygdala, VTA ventral tegmental area, EYFP enhanced yellow fluorescent protein.

We next used Fos-targeted recombination in active population (FosTRAP) mice to distinguish the brain regions associated with anhedonic behavior after CUS. Following the administration of tamoxifen, active CreERT2-expressing cells undergo Cre-mediated recombination (“Trapped”), which results in permanent expression of effector genes^34^. Approximately 16 h after tamoxifen injection, the effect rapidly increases, reaching a maximum at ~24 h before rapidly decreasing after ~36 h, and Fos is no longer labeled^34^. Because Fos is gradually labeled 12 h after tamoxifen injection, we deprived the mice of water for 16 h prior to tamoxifen injection to avoid Fos tagging via the drinking water before the SPT. Sixteen hours after receiving a single i.p. injection of tamoxifen (7.5 µl/g), all the mice were provided water and sucrose solution ad libitum for 7 days (Fig. 2a)^34^. No animals transformed from one behavioral phenotype to another under tamoxifen injection, which was confirmed by conducting the SPT before and after injection (SPT1 vs. SPT2, Fig. 2c). After conducting SPTs in FosTRAP mice exposed to CUS, we counted the number of Fos^+^ cells [Fos^+^ (red)/DAPI^+^ (blue)] in stress- and depression-associated brain regions in the limbic system (Fig. 2b). In several brain regions, such as the nucleus accumbens (NAc), basolateral amygdala (BLA), and ventral tegmental area (VTA), the number of Fos^+^ cells did not significantly differ among the groups. In the mPFC and hippocampus (HPC), the SUS mice possessed a lower number of Fos^+^ cells than the CTRL mice did (Fig. 2d). Unlike those in the HPC, the number of Fos^+^ cells in the mPFC significantly differed between the CTRL and RES mice (Fig. 2d). These data suggest that the mPFC has the greatest association with subphenotypes of CUS-induced anhedonic behaviors (Fig. 2c, d). Our findings are strongly supported by human studies showing that anhedonia in major depressive disorder (MDD) patients is linked to impaired connectivity between the PFC and reward- and emotion-related cognitive regions during the processing of positively valued stimuli^9,35^. Therefore, we focused on the mPFC to elucidate apparent differences in hedonic responses in groups exposed to CUS.

To determine whether the neural activity in the mPFC was associated with CUS-induced anhedonic behavior, we optogenetically manipulated mPFC neurons during the SPT after CUS (i.e., SPT2) using the excitatory channelrhodopsin (ChR2). To this end, AAV5-hSyn-hChR2-EYFP and the control virus were bilaterally injected into the mPFC, and after 1 week, optic fibers were implanted into the virus-infected mPFC (Fig. 2e, f). These procedures generated the following four mouse groups: CUS-exposed mice with ChR2 stimulation (CUS+ChR2), stress-naïve mice with ChR2 stimulation (CTRL+ChR2), CUS-exposed mice without ChR2 stimulation (CUS + EYFP), and stress-naïve mice without ChR2 stimulation (CTRL + EYFP). After 28 days of CUS, we examined the baseline sucrose preference (lights off) and conducted the SPT with phasic stimulation (lights on, five pulses at 20 Hz, 40 ms pulse durations, every 10 s)^36^. The results showed that the preference of the CUS + EYFP and CUS+ChR2 mice for sucrose solution during the first 30 min of the trial without 473 nm blue light exposure was less than that of the stress-naïve groups. Conversely, the sucrose reactivity of the CUS+ChR2 group sharply increased under 473 nm blue light for 30 min; however, the CUS + EYFP group showed no significant increase in sucrose reactivity. Moreover, the stress-naïve CTRL group exhibited high levels of hedonic behavior regardless of optic stimulation (Fig. 2g). Together with the FosTRAP data, these optogenetic data suggest that neuronal activity in the mPFC is associated with the regulation of CUS-induced anhedonic behaviors. Our data are consistent with evidence from previous human studies showing that the PFC is important in anhedonia^9,35^.

### Distinct transcriptional patterns depend on the degree of anhedonia after CUS

**Fig. 3:**
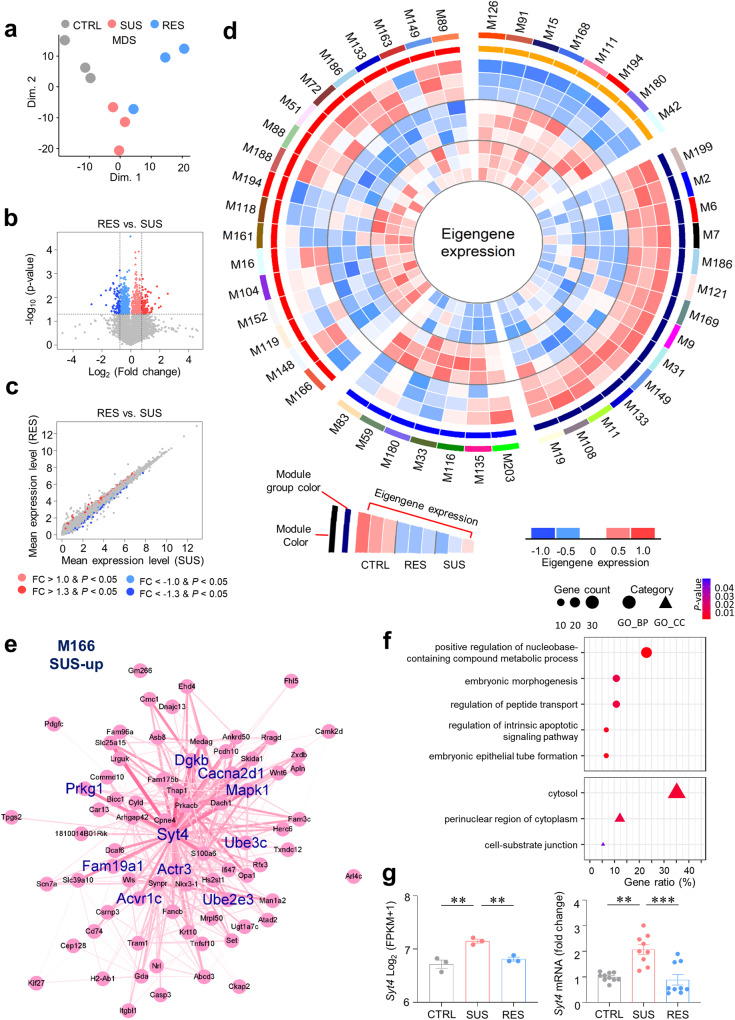
Identification of the behavior phenotype-specific module after CUS. **a** Following CUS, each group was separated and mapped to different dimensions across the whole genome. **b** A volcano plot displaying differential gene expression between the SUS and RES groups. The x-axis represents the log_2_ of the fold change in the gene [log_2_ (fold change)], and the *y*-axis represents the −log_10_ (*p* value). **c** Scatter plot displaying the differential gene expression between the SUS and RES groups. The genes highly expressed in SUS and RES animals are indicated by blue and red data points, respectively, while the genes that were not significantly differentially expressed in either of the groups are represented by gray data points. **d** CUS- or anhedonia susceptibility-related modules from 203 gene coexpression modules constructed via WGCNA. The modules were arranged clockwise from the modules with a large absolute value of the correlation coefficient with sample traits (CUS or anhedonic susceptibility). The module color is indicated by the circle’s outermost color, and the next color refers to the module group (orange: CUS-up, navy: CUS-down, blue: RES-up/SUS-down, red: RES-down/SUS-up). The expression patterns of eigengenes and summaries of the gene expression pattern of each module are colored and displayed in the order of SUS, RES, and CTRL from the inner line of the circle. **e** The intramodular coexpression network of a gene module specifically overexpressed in the SUS group was visualized. The thickness of the line indicates the strength of the correlation. **f** Gene Ontology enrichment analysis of the biological processes and cellular components of SUS-up module genes. **g** *Syt4* expression in the M166 module after RNA sequencing (left, one-way ANOVA with Tukey’s test; *F*_2,6_ = 18.30; *p* = 0.0028; *n* = 3) and qRT‒PCR validation after CUS (right, Kruskal‒Wallis test with Dunn’s test; *H* = 15.32; *p* = 0.0005; *n* = 10, 9, 9). ^**^p < 0.01, ^***^p < 0.001. The bar graphs show the mean ± SEM.

Next, to characterize anhedonia-specific transcriptional modifications in the mPFC, we performed RNA sequencing of mPFC tissue from each mouse that had undergone the SPT after CUS and investigated the anhedonia susceptibility gene network through differentially expressed gene (DEG) analysis and WGCNA. RNA sequencing produced ~54 to 93 million mapped reads for each sample (Supplementary Table 2). The transcriptome in the mPFC region was well clustered into groups according to stress subphenotype (Fig. 3a). DEGs (*p* < 0.05, |fold change | > 1.3) between the CTRL group and each CUS group and between the SUS and RES groups were identified. Although the SUS and RES groups were exposed to the same CUS, only ~20% of the genes in the DEG list overlapped when comparing each group to the CTRL group (Fig. 3b, c*;* Supplementary Fig. 5; and Supplementary Table 3). These data strongly suggest that SUS and RES have significantly different molecular mechanisms.

We next investigated the biological roles of the DEGs through Gene Ontology (GO) and Kyoto Encyclopedia of Genes and Genomes (KEGG) pathway enrichment analyses of six DEG groups (SUS-up, SUS-down, RES-up, RES-down, RES-up/SUS-down, SUS-up/RES-down; Supplementary Fig. 6, and Supplementary Table 4). A comparison of the SUS and CTRL groups led to the identification of various enriched neuronal function-related terms, including “learning,” “negative regulation of synaptic vesicle exocytosis,” “synapse,” and “long-term potentiation” in the SUS-up group and “transsynaptic signaling,” “regulation of neurotransmitter levels,” “axon part,” “neuronal dense core vesicle,” and “axon guidance” in the SUS-down group (Supplementary Fig. 6a, b). Moreover, genes related to “nervous system development,” including “myelination,” were upregulated in the RES group compared to the CTRL group (Supplementary Fig. 6c), and RES-down genes were enriched in terms related to cellular component biogenesis and organization (Supplementary Fig. 6d). Interestingly, genes related to “synapse” and “GDP binding” were commonly enriched in the SUS-up and RES-up groups, while those related to the “HIF-1 signaling pathway” were commonly enriched in the SUS-down and RES-down groups. It is likely that these biological pathways were affected by stress itself independently of subphenotype (Supplementary Fig. 6a–d). Moreover, comparison of the SUS and RES groups revealed that the expression of genes related to nervous system development, synapses, and cognition was relatively high in the RES group (Supplementary Fig. 6e), while the genes related to cell adhesion and cell communication were highly expressed in the SUS group (Supplementary Fig. 6f).

### Coexpression network analysis was used to construct CUS subphenotype-associated gene modules

Using WGCNA to discover behavioral phenotype-specific genes, we identified 203 coexpressed gene modules (Supplementary Table 5). After constructing the gene coexpression modules, we investigated the relationships between the modules and sample traits through linear regression and Pearson correlation (Supplementary Table 6). Linear regression revealed that 43 of the 203 modules were significantly associated with the sample traits (CTRL, RES, and SUS; *p* < 0.05). We also calculated the module–trait relationships through Pearson correlation between the module eigengene and traits (CUS or stress susceptibility) to identify the effects of CUS and stress susceptibility separately. As a result, eight CUS-up (RES-up/SUS-up), 14 CUS-down (RES-down/SUS-down), seven RES-up (SUS-down), and 18 RES-down (SUS-up) modules were selected as CUS- and anhedonia susceptibility-related modules, and their summed eigengene expression was visualized (Fig. 3d). We further characterized the following six representative modules according to their module–trait relationships, module sizes, and expression patterns: M91 (CUS-up), M199 (CUS-down), M166 (SUS-up), M203 (SUS-down), M33 (RES-up), and M104 (RES-down) (Fig. 3e, Supplementary Figs. 7–12). To confirm the biological functions of these modules, we conducted GO and KEGG pathway enrichment analyses for the DEGs (Fig. 3f, Supplementary Figs. 7–12, and Supplementary Table 7). M91, a CUS-up module, was found to be highly expressed in both the RES and SUS groups (Supplementary Fig. 7a) and was enriched in various terms, including “nervous system development” (*p* = 6.7 × 10^-6^), “synapse” (*p* = 1.2 × 10^-6^), and “oxytocin signaling pathway” (*p* = 1.3 × 10^-2^; Supplementary Fig. 7c, d). M166, an SUS-up module, was enriched in the terms “regulation of peptide transport” (*p* = 1.0 × 10^-2^) and “regulation of intrinsic apoptotic signaling pathway” (*p* = 3.8 × 10^-3^; Fig. 3f and Supplementary Fig. 8a). M33 was upregulated in the RES group compared to the other groups (Supplementary Fig. 9a), and it was associated with the biological processes of “positive regulation of transcription, DNA-templated” (*p* = 4.1 × 10^-3^) and “axo-dendritic transport” (*p* = 6.3 × 10^-4^; Supplementary Fig. 9c). M199 was downregulated in both the RES and SUS groups compared to the CTRL group (Supplementary Fig. 10a). M199 was associated with the biological process of RNA metabolism (Supplementary Fig. 10c), while M203, the expression of which was downregulated only in SUS (Supplementary Fig. 11a), was related to “nervous system development” (*p* = 5.2 × 10^-3^) and “presynapse” (*p* = 1.6 × 10^-3^; Supplementary Fig. 11c). Conversely, the M104 module was downregulated only in the RES group (Supplementary Fig. 12a) and was likely related to cell death and metal ions (Supplementary Fig. 12c).

To investigate the cause of anhedonia susceptibility, we focused on investigating M166, which was identified as the module most highly correlated with anhedonia susceptibility (Fig. 3d and Supplementary Table 6). We found that M166 expression increased only in anhedonia-susceptible mice (Supplementary Fig. 7a). In M166, which comprises a network of 77 genes, *Syt4* was a hub gene coexpressed with 70 (92%) other genes within the module, whereas other hub genes in the module were coexpressed with fewer genes (11–52 genes, 14%–68%) (Fig. 3e and Supplementary Table 5). Therefore, *Syt4*, which had the largest gene network, likely plays a central role in this module. *Syt4* was also a DEG in the SUS-up group and was found to be associated with biological processes, including “intracellular transport” (*p* = 1.8 × 10^-6^), “negative regulation of synaptic vesicle exocytosis” (*p* = 1.4 × 10^-5^), and “synapse” (*p* = 2.2 × 10^-4^; Supplementary Fig. 6a and Supplementary Table 4). Additionally, when we validated the sequencing data via qRT‒PCR, *Syt4* expression increased only in SUS mice, similar to the results of RNA sequencing (Fig. 3g). Taken together, these findings suggest that *Syt4* plays a significant regulatory role in the anhedonia sensitivity network.

### Localization and activity of Syt4 in the mPFC

**Fig. 4:**
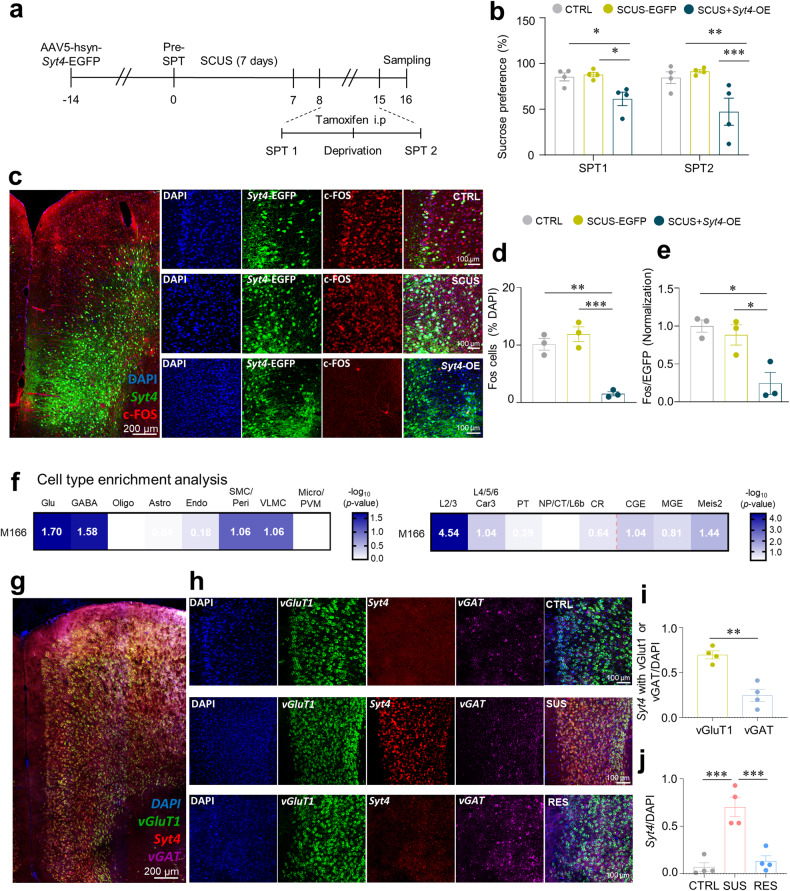
Effects of Syt4 on mPFC activity and cell type/layer-specific localization. **a** Experimental procedures for capturing activated neurons during anhedonic behaviors. **b** Sucrose preference before (SPT1) and after i.p. injection of tamoxifen (SPT2; two-way ANOVA with Fisher’s LSD test: group effect, *F*_2,18_ = 12.82, *p* = 0.0003; time effect, *F*_1,18_ = 0.3658, *p* = 0.5528; interaction, *F*_2,18_ = 0.7190, *p* = 0.5007, *n* = 4). **c** Representative images of Fos^+^ cells induced by *Syt4* overexpression (*Syt4-*OE) and SCUS. **d** The percentage of Fos+ cells relative to DAPI-stained cells in each group following SCUS (one-way ANOVA with Tukey’s test, *F*_2,6_ = 32.57, *p* = 0.0006, *n* = 3, 3, 3). **e** The percentage of Fos+ cells relative to EGFP^+^ cells in each group following SCUS (one-way ANOVA with Tukey’s test, *F*_2,6_ = 10,94 *p* = 0.0100, *n* = 3, 3, 3). **f** Cell-type enrichment analysis of the M166 module, which includes Syt4 as a hub gene (Fisher’s exact test). **g** A representative image of fluorescence in situ hybridization for *Syt4* localization. **h** Representative images depicting the expression of *Syt4*, vGluT1, and vGAT in the PFC were captured for each group. **i** Cell-type specificity of *Syt4* expression in the PFC (unpaired t test, t_6_ = 5.548, *p* = 0.0014, *n* = 4). **j** *Syt4* expression in the PFC according to CUS phenotype (one-way ANOVA with Tukey’s test, *F*_2,9_ = 23.83, *p* = 0.0003, *n* = 4). ^*^p < 0.05, ^**^p < 0.01, ^***^p < 0.001. The bar gra*p*hs show the mean ± SEM. Glu glutamatergic neuron, GABA GABAergic neuron, Oligo oligodendrocyte, Astro astrocyte, Endo endothelial cell, SMC/Peri smooth muscle cell/pericyte, VLMC vascular and leptomeningeal cell, Micro/PVM microglia/perivascular macrophage, L2/3 layer 2/3 glutamatergic neuron, L4/5/6 Car3 layer 4/5/6 or Car3^+^ glutamatergic neuron, PT pyramidal tract glutamatergic neuron, NP/CT/L6b near-projecting/corticothalamic/layer 6b glutamatergic neuron, CR Cajal-Retzius glutamatergic neuron, CGE caudal ganglionic eminence-originated GABAergic neuron, MGE medial ganglionic eminence-originated GABAergic neuron, Meis2 Meis2^+^ GABAergic neuron.

We observed a notable decrease in mPFC activity in SUS mice (Fig. 2d). On the basis of our sequencing findings highlighting *Syt4* as a key gene linked to the development of an SUS phenotype, we further explored the relationship between *Syt4* and mPFC activity. To achieve this goal, we introduced the AAV5-hsyn-*Syt4*-EGFP virus, which causes *Syt4* overexpression (*Syt4*-OE), into the mPFC of Fos-Trap x Ai9 mice (Fig. 4a) and confirmed successful induction of *Syt4* expression by the virus (Supplementary Fig. 14a, b). Interestingly, the *Syt4*-OE group exhibited pronounced anhedonia even after exposure to only 1 week of subthreshold chronic unpredictable stress (SCUS) (Fig. 4b). To examine whether SCUS influenced mPFC activity, Fos+ cells were labeled with tamoxifen after SPT1 (Fig. 4c). As with the SPT, a marked reduction in mPFC activity was observed in the *Syt4*-OE animals under SCUS conditions (Fig. 4d, e).

Next, we compiled a list of cell-type marker genes for cell-type enrichment analysis using single-cell RNA sequencing data from the Allen Brain Map site for mouse brains^37^. The results showed that the genes within module M166 were predominantly expressed in glutamatergic and GABAergic neurons (Fig. 4f). Further exploration revealed that the M166 module genes were relatively distributed in layer 2/3 of the PFC (Fig. 4f). To validate the results of the cell type enrichment analysis, we conducted FISH. Our FISH results indicated the predominant presence of *Syt4* in excitatory neurons (vGluT1) rather than in inhibitory neurons (vGAT) (Fig. 4g–j). Additionally, we confirmed the abundant expression of *Syt4* in the PFC of SUS mice, which was consistent with the sequencing data (Fig. 4j). To explore the layer-specific expression of *Syt4*, we conducted experiments using *Wfs1*-Cre (layer 2/3) and *RBP4*-Cre (layer 5) mice. To achieve layer-specific labeling, we injected the AAV5-hsyn-DIO-mCherry virus into the PFC of layer-Cre mice (Supplementary Fig. 13a–c). We observed a significant increase in *Syt4* expression in both layer 2/3 and layer 5 of CUS mice compared to that in CTRL mice (Supplementary Fig. 13d–g). Although there was a slight tendency for higher *Syt4* expression in layer 2/3 of the CUS mice than in layer 5, the difference was not significant (Supplementary Fig. 13h). These findings collectively indicate the abundant expression of *Syt4* in the PFC of SUS mice, particularly in excitatory neurons.

### Pro-susceptible role of SYT4 in anhedonic responses

**Fig. 5:**
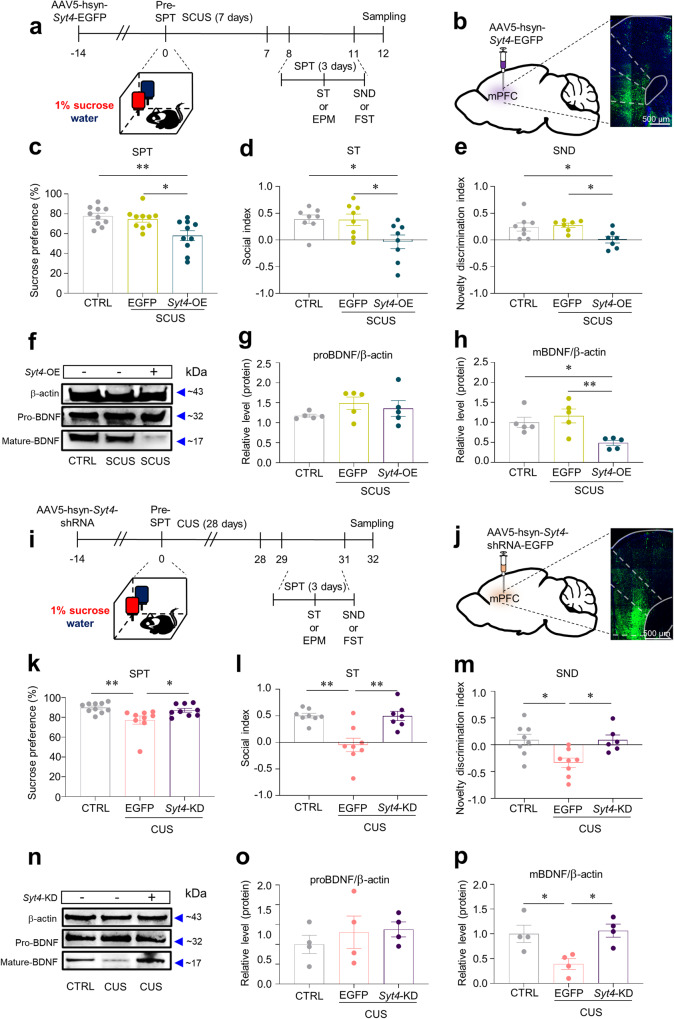
Synaptotagmin-4 (SYT4) in the mPFC plays a critical role in anhedonic behaviors. **a** Experimental procedures to investigate whether *Syt4*-OE induces pro-depressive behaviors. **b** A confocal image showing the injection sites of AAV5-hsyn-Syt4-EGFP into the mPFC. **c**–**e** After SCUS, *Syt4*-OE in the mPFC significantly induced anhedonic behaviors such as impaired sucrose preference (**c** one-way ANOVA with Tukey’s test, *F*_2,27_ = 7.639, *p* = 0.0023, *n* = 10, 10, 10), defective sociability (**d** one-way ANOVA with Tukey’s test, *F*_2,21_ = 5.094, *p* = 0.0157, *n* = 8, 8, 8), and impaired preference for social novelty (**e** one-way ANOVA with Tukey’s test, *F*_2,19_ = 5.325, *p* = 0.0146, *n* = 8, 7, 7). **f**, **n** Representative western blotting of BDNF. **g** Expression of proBDNF among groups (one-way ANOVA with Tukey’s test, *F*_2,12_ = 1.1137, *p* = 0.3531, *n* = 5). **h** Expression of mature BDNF in the groups (one-way ANOVA with Tukey’s test, *F*_2,12_ = 7.395, *p* = 0.0081, *n* = 5). **i** Experimental procedures to investigate whether *Syt4*-KD blocks depressive-like behaviors. **j** A confocal image showing the injection sites of AAV5-hsyn-Syt4-shRNA-EGFP into the mPFC. **k**–**m** *Syt4*-KD prevented CUS-induced anhedonic behaviors. **k** SPT: Kruskal–Wallis test with Dunn’s test (*H* = 10.97, *p* = 0.0041, *n* = 10, 9, 9); **l** ST: one-way ANOVA with Welch’s test (*W*_2,11.13_ = 8.074, *p* = 0.0068, *n* = 8, 8, 7); and **m** SND test: one-way ANOVA with Tukey’s test (*F*_2,19_ = 6.675, *p* = 0.0064, *n* = 8, 7, 6). **o** Expression of proBDNF among groups (one-way ANOVA with Tukey’s test, *F*_2,9_ = 0.4698, *p* = 0.6396, *n* = 4). **p** Expression of mature BDNF in the groups (one-way ANOVA with Tukey’s test, *F*_2,9_ = 6.947, *p* = 0.00150, *n* = 4). *p < 0.05, **p < 0.01. The bar graphs show the mean ± SEM. EGFP enhanced green fluorescent protein.

To establish the causal link between SYT4 and anhedonic behavior, we administered AAV5-hSyn-*Syt4*-EGFP into the mPFC of mice 2 weeks prior to stress exposure (Fig. 5a, b). A pre-SPT, conducted prior to stress induction, aimed to assess the viral impact in the absence of stress. The results revealed no group differences in pleasure-seeking behavior (Supplementary Fig. 14a–d). We next performed SCUS to investigate whether *Syt4*-OE elicited strong anhedonic behavior even under SCUS conditions, which did not induce any behavioral effects (Fig. 5a). We observed that the SPT, ST, and SND test results did not significantly differ between the CTRL and SCUS + EGFP mice. Conversely, *Syt4*-OE with SCUS (SCUS+*Syt4*-OE) resulted in a significant reduction in sucrose preference (Fig. 5c). Moreover, the SCUS+*Syt4*-OE mice exhibited decreased social interaction with the enclosure containing a stimulus mouse (Fig. 5d) and a reduced ability to recognize a novel mouse (Fig. 5e), with no significant change in the total distance traveled (Supplementary Fig. 14e, f). To further evaluate the effects of *Syt4*-OE and/or SCUS on despair behavior, we performed the FST in a different cohort. The SCUS + EGFP and SCUS+*Syt4*-OE mice exhibited significantly greater immobility time in the FST than did the CTRL mice, with the SCUS+*Syt4*-OE mice displaying greater immobility than the CTRL and SCUS + EGFP mice (Supplementary Fig. 14g). Given the lack of change in locomotor activity, these findings suggest that *Syt4*-OE promotes depressive-like behaviors under subthreshold CUS.

We then investigated whether the inhibition of *Syt4* expression (*Syt4*-KD) (Supplementary Fig. 15a–c) during CUS led to an improved pursuit of pleasure and/or reduced despair- and anxiety-like behaviors. Two weeks before CUS, we bilaterally injected AAV5-hSyn-*Syt4*-shRNA-EGFP into the mPFC (Fig. 5i, j). Before CUS, we performed the pre-SPT to assess whether the virus influenced pleasure-seeking behavior. The results revealed no significant change in sucrose preference among the groups (Supplementary Fig. 15d). To further measure depressive-like behavior following the CUS paradigm, we performed the SPT, ST, and SND test. The CUS + EGFP mice showed a marked decrease in sucrose preference following CUS compared to the CTRL and CUS+*Syt4*-KD mice (Fig. 5k). Similarly, *Syt4* KD blocked the detrimental effects of CUS on social interactions with a stimulus mouse in the ST, as well as the recognition of a novel mouse in the SND test (Fig. 5l, m), with no significant change in total distance traveled (Supplementary Fig. 15e, f). We also tested whether selective *Syt4* KD in the mPFC regulated CUS-induced despair behaviors by conducting the FST on mice from different cohorts post-CUS. As expected, CUS increased despair-like behavior, which was prevented by *Syt4* KD in the mPFC (Supplementary Fig. 15g). Overall, our evidence indicated that the modulation of *Syt4* expression in stressful settings was strongly associated with depression-like behaviors, including physical and social anhedonia.

### SYT4 participates in the BDNF–TrkB system to induce depressive-like behavior

SYT4 is a negative regulator of the secretion of various neurotrophins and neurotransmitters^25,37,38^. In particular, SYT4 is an important factor in the release of BDNF^25,39^ and dopamine;^40,41^ signaling in both regions of the mPFC has been linked to depressive-like behaviors. Therefore, we initially conducted BDNF western blotting assays using *Syt4*-OE and *Syt4*-KD samples to explore the link between *Syt4* and BDNF. Consistent with the findings of prior studies^25,39^, we confirmed that *Syt4* negatively regulates mature BDNF (mBDNF) in both *Syt4*-OE (Fig. 5f, h) and *Syt4*-KD animals (Fig. 5n, p): *Syt4*-OE decreased mBDNF protein levels in the mPFC of SCUS animals, while *Syt4*-KD restored the CUS-induced decrease in mBDNF levels. However, no such effects of *Syt4* were observed on proBDNF, the immature form of BDNF (Fig. 5f, g, n, o). Additionally, we found no significant alteration in dopamine levels attributed to *Syt4* manipulation (Supplementary Fig. 16g, h). These findings suggest that SYT4 exerts pro-susceptible effects on depressive-like behaviors through the regulation of BDNF signaling but not proBDNF or dopamine within the mPFC.

**Fig. 6:**
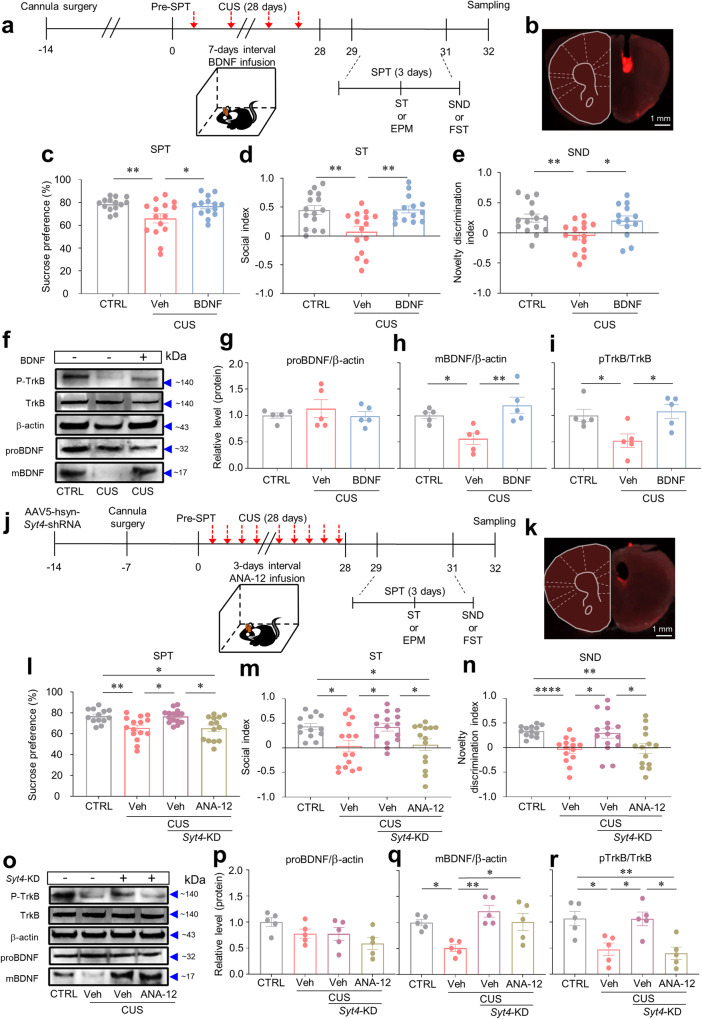
SYT4 mediates anhedonia by regulating BDNF and TrkB signaling. **a** Experimental procedures to investigate whether BDNF release, which is regulated by *Syt4*, has a proresilient effect on CUS-induced anhedonic behaviors. **b** A representative image of the cannula implantation sites in the mPFC. **c**–**e** Intra-mPFC infusion of BDNF prevented CUS-induced anhedonic behaviors. **c** SPT: one-way ANOVA with Welch’s test (*W*_2,24.27_ = 4.168, *p* = 0.0278, *n* = 14, 15, 14); **d** ST: one-way ANOVA with Tukey’s test (*F*_2,41_ = 7.912, *p* = 0.0012, *n* = 15, 15, 14); and **e** SND test: one-way ANOVA with Tukey’s test (*F*_2,40_ = 5.728, *p* = 0.0065, *n* = 15, 15, 13). **f**, **o** Representative western blotting of BDNF and TrkB. **g** Expression of proBDNF among groups (one-way ANOVA with Tukey’s test, *F*_2,12_ = 0.4653, *p* = 0.6388, *n* = 5). **h** Expression of mature BDNF in the groups (one-way ANOVA with Tukey’s test, *F*_2,12_ = 0.4653, *p* = 0.6388, *n* = 5). **i** Relative proportion of phospho-TrkB to total TrkB among groups (one-way ANOVA with Tukey’s test, *F*_2,12_ = 5.656, *p* = 0.0186, *n* = 5). **j** Experimental procedure for investigating whether the mediating role of *Syt4* in CUS-induced anhedonic behaviors is regulated by BDNF–TrkB signaling. **k** A representative image of the cannula implantation sites in the mPFC. **l**–**n** *Syt4*-KD blocked CUS-induced anhedonia, and the reversal effect of *Syt4*-KD on depressive behaviors was prevented by ANA-12. **l** SPT: one-way ANOVA with Welch’s test (*W*_3,28.62_ = 7.035, *p* = 0.0011, *n* = 13, 15, 15, 14); **m** ST: one-way ANOVA with Welch’s test (*W*_2,11.13_ = 8.074, *p* = 0.0068, *n* = 13, 15, 15, 14); **n** SND test: Kruskal–Wallis test with Dunn’s test (*H* = 10.10, *p* = 0.0177, *n* = 13, 15, 15, 14). **p** Expression of proBDNF among groups (one-way ANOVA with Tukey’s test, *F*_3,16_ = 2.623, *p* = 0.0862, *n* = 5). **q** Expression of mature BDNF in the groups (one-way ANOVA with Tukey’s test, *F*_3,16_ = 7.546, *p* = 0.0023, *n* = 5). **r** Relative expression of phospho-TrkB to total TrkB among groups (one-way ANOVA with Tukey’s test, *F*_3,16_ = 8.076, *p* = 0.0017, *n* = 5). ^*^p < 0.05, ^**^p < 0.01, ^****^p < 0.0001. The bar graphs show the mean ± SEM.

To investigate whether interactions between SYT4 and BDNF are associated with stress-related depressive behaviors, we pharmacologically manipulated BDNF signaling in the mPFC during CUS. Two weeks after cannula implantation in the mPFC, we infused BDNF or vehicle every 7 days during CUS exposure (Fig. 6a, b). The results showed that pharmacological intervention with BDNF during CUS effectively mitigated the adverse effects of CUS on anhedonia and novel recognition without altering the total distance traveled (Fig. 6c–e, Supplementary Fig. 16a, b). Similarly, intra-mPFC BDNF infusion reversed CUS-induced despair-like behavior in the FST (Supplementary Fig. 16c). We then examined whether the reversal effects of intra-mPFC BDNF infusion on CUS-induced despair-like behaviors were mediated through activated TrkB, which acts as a specific receptor for mBDNF^42,43^. The results showed that intra-mPFC BDNF infusion blocked the reduction in mBDNF but not proBDNF induced by CUS. Consistently, the level of activated TrkB, which was estimated by the ratio of phosphorylated TrkB to total TrkB, was decreased by CUS, and this effect was reversed by intra-mPFC BDNF infusion (Fig. 6f–i). These data suggest that BDNF–TrkB signaling in the mPFC has an antidepressant-like role in CUS-induced depressive-like behaviors. To further investigate the putative mediating role of BDNF–TrkB signaling in the pro-susceptible effect of SYT4 on anhedonic behaviors, we suppressed *Syt4* expression in the mPFC and simultaneously blocked BDNF–TrkB signaling by infusing ANA-12, a TrkB antagonist, into the mPFC (Fig. 6j, k). As shown in Fig. 5k–m, CUS-induced depressive-like behaviors were reversed by *Syt4*-KD. Periodic administration of ANA-12 blocked the reversal of depressive-like behaviors by *Syt4*-KD in the SPT, ST, SND test, and FST (Fig. 6l–n, Supplementary Fig. 16d–f). These data suggest that BDNF-TrkB signaling mediates the proresilient effect of *Syt4*-KD in the SPT, ST, SND test, and FST.

We also investigated the impact of *Syt4-*KD and ANA-12 on BDNF and TrkB expression through western blot analysis. Our results confirmed that Syt4-KD significantly increased mBDNF expression (Fig. 6o–q). Interestingly, in the group infused with ANA-12, although the mBDNF levels were elevated by *Syt4*-KD, TrkB activity was suppressed by ANA-12, resulting in anhedonic symptoms (Fig. 6l–r). Taken together, our findings suggest that increased *Syt4* expression in the mPFC under stress conditions diminishes the activity of the BDNF-TrkB system, leading to anhedonic behavior (Supplementary Fig. 17).

## Discussion

Anhedonia is considered a core feature of depression, although its symptoms are heterogeneous in patients with MDD. Anhedonia can be assessed in terms of various reward-related behavioral responses, which often include social interaction because social motivation/reward can be a powerful driver of human and animal behaviors^44^. In the present study, we evaluated anhedonic behaviors by measuring social anhedonia using the sociability test and physical anhedonia using the SPT. Analysis of covariance and PCA of the outcomes of various tests for depressive- and anxiety-like behaviors revealed that physical anhedonia (i.e., sucrose preference) was more strongly associated with social competence, social novelty recognition, and despair behavior than with anxiety-like behavior.

We aimed to identify the most salient and prominent depressive traits in the CUS model. Analysis of covariance and PCA revealed that anhedonia following CUS was the most distinct depressive behavioral trait and served as the central hub of other depressive-like behaviors. However, these findings were not unexpected given that anhedonic behavior has long been considered a representative behavioral outcome in the CUS model^45^ Notably, there was no significant correlation observed between the ST and SND test. In the most recent study, the SND test was found to share similar features with contextual memory, indicating that it operates via distinct mechanisms from the ST^46^. Another study demonstrated that the ensembles of neurons within the PFC differ between sociability and social novelty preference^47^. These findings suggest distinct neural mechanisms specific to the ST or SND test, although both are largely influenced by anhedonia^47^.

Based on our multivariate analysis and *K*-means clustering data, anhedonia was identified as the most general test for estimating individual variations in CUS-induced depressive-like behaviors. However, few studies have analyzed the transcriptomes of anhedonia, focusing instead on individual differences. To investigate the molecular mechanism underlying the anhedonic responses, we performed WGCNA with RNA sequencing data considering the behavioral subphenotypes based on sucrose preference (anhedonic [susceptible] vs. nonanhedonic [resilient]) following CUS, which is an etiologically validated model of depression in rodents. Our data demonstrated that CUS induces the expression of *Syt4*, the potent driver gene of M166, which is the coexpression module most associated with the anhedonic behavioral phenotype, in the mPFC. Interestingly, previous studies have revealed that *SYT4* expression is increased in the frontal cortex of female and male patients with MDD^48,49^. Furthermore, a previous preclinical study demonstrated that *Syt4*(–/–) mice exhibit reduced anxiety and depressive-like behavior^50^. Consistent with these findings, our data showed that virus-mediated *Syt4* overexpression in the mPFC elicits pro-susceptible effects on depressive-like and anxiety-like behaviors, including anhedonic behaviors. Overall, our findings suggest that CUS-induced SYT4 expression promotes stress susceptibility.

In the present study, we observed that the overexpression of *Syt4*, which is associated with presynaptic docking or vesicle fusion and release in an activity-dependent manner^25,26,39^, led to a decrease in the protein levels of mBDNF but not proBDNF in the mPFC. Furthermore, the reduction in BDNF induced by CUS was reversed by the virus-mediated knockdown of *Syt4* in the mPFC. Similar to the expression patterns of BDNF, the CUS-induced decrease in TrkB activation, which results from the release and binding of BDNF^51,52^, in the mPFC was also blocked by *Syt4* knockdown. Both previous research and our own study suggest that CUS-induced *Syt4* may play a negative regulatory role in BDNF-TrkB signaling within the mPFC.

Our study has limitations that warrant discussion. We found that the pro-susceptible effects of SYT4 involved reducing BDNF-TrkB signaling in the mPFC. Notably, *Syt4* has also been associated with other neurotransmitters, including dopamine and oxytocin^37,40,41,53^. Although disrupted dopamine signaling in the mPFC is often linked to depressive-like behaviors^54–56^, our ELISA results did not reveal significant effects of *Syt4* manipulation on PFC dopamine levels (Supplementary Fig. 16g, h). Although oxytocin is known to be relevant to anxiety^57^, we focused primarily on anhedonia when identifying *Syt4* through RNA sequencing. Although we conducted EPM tests to assess anxiety symptoms, the results showed a weak correlation with anhedonia (Fig. 1m and Supplementary Fig. 2). As a result, we did not extensively investigate oxytocin. Future research could explore the potential relationship between Syt4 and oxytocin in the context of anxiety behaviors.

Although our manipulation of *Syt4* and stress altered BDNF protein levels and behaviors, the mechanisms underlying SYT4-mediated regulation of BDNF release and TrkB activation and its downstream effects under chronic stress conditions have not been fully elucidated. Previous studies have demonstrated that SYT4, which is present in BDNF-containing vesicles within cultured hippocampal neurons, governs depolarization-induced BDNF release^25,26,39^. Furthermore, SYT4 has been linked to adjusting synaptic function and plasticity (e.g., long-term potentiation) through BDNF release^25,26,39^. Indeed, a previous study reported the results of a coculture assay with hippocampal neurons, which showed an increase in presynaptic strength only in terminals contacting *Syt4* knockout neurons, which increased BDNF release^25^. However, given that most research on SYT4–BDNF has focused on hippocampal neurons, future studies should determine whether the same synaptic function occurs in the mPFC after CUS.

The sex-specific action of SYT4-BDNF in depression requires further investigation. Preclinical studies have shown that ovariectomy (OVX) of female rodents significantly decreases BDNF protein levels in the HPC, inducing depressive-like behaviors, which are restored by 17β-estradiol (a potent estrogen)^58,59^. Other studies have shown that 17β-estradiol decreases *Syt4* expression in primary cultured hippocampal neurons^60^ and in the hypothalamus of ovariectomized mice^61^. These data suggest that estrogen deficiency or fluctuations are closely associated with the effect of SYT4-BDNF on the pathophysiology of depression in females. Indeed, a large-scale analysis of the transcriptional organization of the human brain revealed that SYT4 is highly expressed in the frontal lobe of female patients with depression but not in that of their male counterparts^48^. However, further studies are needed to investigate the relationships among SYT4, BDNF, and sex hormones in female patients with depression to develop optimized precision interventions for treating depression, especially for females, who have a greater lifetime incidence of depression than males.

Using coexpression network analysis, we confirmed that the susceptible and resilient mice exhibited dramatically distinct biological processes (Fig. 3 and Supplementary Figs. 5–12). We also found several intriguing hub genes in M166. For example, *mitogen-activated protein kinase* 1 (*Mapk1*) is associated with the initiation and progression of inflammatory processes, which are related to bipolar disorder and MDD, in several human brain regions^62,63^. Previous studies have shown that *Mapk1*-encoded protein expression is increased in the PFC in the Flinders sensitive line (FSL), a genetic rat model of depression^64^. Moreover, *CACNA2D1*, which has been detected in human genome-wide association data as a candidate gene for depression^65^ and bipolar disorder^66^, has been suggested to be a potential target for depression treatment and rapid antidepressant effects^67^. However, further research should be performed to address this potential. Moreover, a genome-wide association study on posttraumatic stress disorder identified *cGMP-dependent protein kinase I* (*PRKG1*) as a risk locus in a military cohort, supporting its role in stress response-related traits in humans^68^. However, how these hub genes interact with each other in the module and influence depressive-like behaviors remains to be elucidated.

In conclusion, we describe the regulatory role of SYT4 in BDNF signaling in the mPFC, which concomitantly results in depressive-like phenotypes in response to chronic stress. Our data suggest that the mPFC SYT4 expression mediates depressive-like behaviors elicited by chronic stress through disruption of BDNF–TrkB signaling in the mPFC. These findings confirm the molecular mechanisms involved in stress susceptibility, particularly anhedonia, and provide a molecular genetic basis for enhancing the understanding of the heterogeneous symptoms of individuals with MDD.

### Supplementary information

Supplementary information

Supplementary table 3

Supplementary table 4

Supplementary table 5

Supplementary table 6

Supplementary table 7
