# Supplementary material for: Synaptotagmin-4 induces anhedonic responses to chronic stress via BDNF signaling in the medial prefrontal cortex
Source: Exp Mol Med. 2024 Feb 1;56(2):329–43. doi: 10.1038/s12276-024-01156-8 (PMC10907712; doi:10.1038/s12276-024-01156-8)
Supplement: Supplementary file 1 — Supplementary information [file 12276_2024_1156_MOESM1_ESM.pdf]

Supplementary Information

Synaptotagmin-4 induces anhedonic responses to chronic stress via BDNF signaling in the medial prefrontal cortex

Jeongseop Kim[1,2,4], Sihwan Seol[3,4], Tae-Eun Kim[1,2], Joonhee Lee[3], Ja Wook Koo[1,2]*, and Hyo Jung Kang[3]*

*Correspondence to: Ja Wook Koo and Hyo Jung Kang

E-mail: (jawook.koo@kbri.re.kr) and (hyokang@cau.ac.kr)

Table of contents:

Materials and Methods

Supplementary Figures 1 to 17

Supplementary Tables 1 to 7

SI References

Materials and Methods

Experimental Animals

In this study, we used 8-to-13-week-old C57BL/6N male mice (25–30 g, Orient Bio, South Korea), heterozygous *Fos*-CreERT mice (JAX strain: 021882), Ai9 mice (JAX strain: 007909), *Wfs1*-CreER mice (layer 2/3, JAX strain: 009103), and *Rbp4*-Cre mice (layer 5, MMRRC strain: 031125-UCD) with a C57BL/6J genetic background. *Fos*-CreERT, Ai9, and *Wfs1*-CreER mice were purchased from The Jackson Laboratory and *RBP4*-Cre mice were acquired from Mutant Mouse Resource & Research Centers (MMRRC) and bred at the Korea Brain Research Institute (KBRI). All mice were exposed to vivarium conditions for at least 1 week before behavioral treatments. The vivarium was set at approximately 23–25°C, with a 12-h light/dark cycle (lights on between 8:00 A.M. and 8:00 P.M.), and the mice were provided *ad libitum* access to food and water. The Institutional Animal Care and Use Committee (IACUC) authorized all operations, which met the animal care criteria set out by the KBRI guidelines (M1-IACUC-19-0009). Every effort was made to minimize the number of animals used in our research and reduce animal suffering.

Chronic unpredictable stress (CUS) and subthreshold unpredictable stress (SCUS)

CUS and SCUS animal models were employed, as previously described[1]. These models are based on the fundamental premise that prolonged stressor exposure is a major contributor to the development of depression in humans and animals. To induce CUS, each animal was subjected to a daily randomized set of minor stressors. Before behavioral assessment, participants were exposed to two or three stressors every day for 4 weeks. The mice were exposed to one stressor during the day and another stressor during the night. To keep the subjects unpredictable to the next stressors, common stressors that have been well-validated

and authorized were chosen randomly from the list shown in Supplementary Table 1. For SCUS, all mice were subjected to two or three daily stimuli for 1 week. The sucrose preference test was conducted for 3 days, beginning the day after the completion of stress. One evaluation was conducted per day, starting from the behavioral experiment with a lower stress. During the CUS experiment, all mice, including controls, were housed four per cage. Control mice, which were subjected to gentle handling, were housed in the home cage until the end of the CUS. All animals were single housed immediately after the end of the 28-day CUS experiment.

Sucrose preference test (SPT)

In the pre-SPT before CUS, the mice were habituated to a two-bottle lid and sucrose solution for 1 day. After 16 h of water deprivation, pre-SPT was performed for 24 h. However, with the exception of the Fos-TRAP experiments, the post-SPT did not proceed with water deprivation. After CUS, three sessions of post-SPT were conducted over 3 days (i.e., one session per day), with one bottle containing a 1% sucrose solution and the other containing water[3]. After 24 h, the position of the bottle was moved to avoid a potential preference for orientation. The bottles were weighed at 10:00 A.M. every day for 3 consecutive days to determine how much sucrose solution (Bottle A) or water (Bottle B) had been drunk. The mice were deprived from water and sucrose solution until 4:30 P.M, when another behavioral experiment (e.g., sociability test [ST], social novelty discrimination [SND] test, forced swim test [FST], or elevated plus maze [EPM) test) was completed. The proportion of sucrose solution drunk relative to the total amount of liquid consumed was used to compute the preference for the sucrose solution, which was measured as a three-session average percentage [weighted sucrose bottle / total weighted] taken over the 3 days of testing. All mice were habituated prior to the SPT test. We performed a ST or EPM test on day 2, and a SND test or FST on day 3 of SPT, as applicable.

Forced swim test (FST)

A FST was conducted according to previously published protocols, with slight modifications[2]. Before the FST test, all mice were placed in a 2000-mL Pyrex glass beaker containing 1200 mL of water for 6 min, and the water was changed between subjects. A video camera mounted on the side of the cylinders was used to capture all of the test sessions. An observer who was unaware of the genotype evaluated and rated the videotapes. Immobility was assessed during the last 4 min of the test.

Elevated plus maze (EPM)

The EPM was designed in a gray plexiglass (arms, 33 cm × 5 cm, with 25-cm high walls on the closed arms). Before the test, all mice were habituated to a room under red light (< 10 lux) for at least 1 h. All mice were positioned in the center of the maze, and their activity was recorded for 5 min under dim illumination[4]. The EPM was washed between the subjects with odorless 70% ETOH. The time spent in each arm, and the locomotion and velocity were measured by the video tracking program SMART3.0.

Novelty suppressed feeding (NSF)

The NSF test was adapted from previous protocols[1]. The mice were deprived of food for at least 12 h before the test was conducted in their home cages. On the test day, the mice were positioned in an open field box (40 cm × 40 cm × 40 cm) with numerous small food pellets placed on a piece of white paper (10 cm × 10 cm) in the center. Each mouse was initially placed in a corner of the cage. The critical test criterion was the time it took to approach the meal and start eating (maximum time: 5 min). To exclude the possibility that stress altered normal appetite and eating, food consumed for the first 5 min was measured immediately after each mouse was returned to its home cage.

Three-chamber sociability and novelty test

Before the test, all mice were habituated to a room under red light (< 10 lux) for at least 1 h. Each experimental mouse was placed in a (57 × 22.5 × 30.5 cm) room with four transparent plexiglass walls and grey plastic flooring, based on the three-chamber task designed previously[3]. The mice could travel freely between the left and right compartments (each 24.5 × 22.5 cm) and the smaller center compartment within the room (8 × 22.5 cm). Each compartment had an inverted wire cup on the left and right. On the first day, the mice were habituated to the three-chamber itself for 5 min. Afterwards, mice that had been bred together at the same age were placed in an enclosure in one room to habituate them for 5 min ("familiar" mice). On the morning of the test, the test mice were first subjected to a 5-min habituation period to acquaint them with the apparatus. Following habituation, the test mouse was contained within the center compartment for 1–2 min while a familiar mouse was placed under one of the two inverted wire cups. Subsequently, the control or CUS mice were allowed to freely explore the chamber for 5 min. The three-chamber task was conducted to test for novelty preference (as described above for sociability); instead of a familiar mouse, a "novel" mouse (a mouse of a similar age that had not been introduced previously) was placed under one of the two inverted cups. The social preference index (I_{SP}) was obtained as follows: I_{SP} = (time in social (or novel) − time in non-social (or familiar)) / (time in social (or novel) + time in non-social (or familiar)).

Stereotaxic surgery, cannula implantation, and micro-infusions

Mice were anesthetized with ketamine and rompun (100 mg/kg; 10 mg/kg) and placed on a stereotaxic instrument (Stoelting). An ophthalmic ointment was applied during stereotaxic

surgery to prevent the eyes from drying out. For *Syt4* manipulation in the PFC, consisting of the prelimbic and infralimbic subareas, adeno-associated virus vector 5 (AAV5) expressing enhanced green fluorescent protein (EGFP) was bilaterally injected under the human synapsin (hSyn) promoter with *Syt4* (i.e., AAV5-hSyn-*Syt4*-EGFP and AAV-hSyn-*Syt4*-shRNA-EGFP – custom made and purchased from ABM, Richmond, Canada). For layer-specific manipulation, we injected AAV5-hsyn-DIO-mCherry (50459-AAV5, Addgene, Massachusetts, USA) into PFC of *Wfs1*-Cre (layer2/3) and *Rbp4*-Cre (layer5) mice. All viruses were bilaterally injected (0.1 µl/min, a total of 0.5 µl/side) into the PFC (AP: + 1.7 mm; ML: ± 0.75 mm; DV: –2.5 mm from the bregma; 15° angle). For optogenetic manipulation in the mPFC, AAV5-hSyn-hChR2(H134R)-EYFP or AAV5-hSyn-EYFP was bilaterally injected into the mPFC (26973-AAV5, Addgene, Massachusetts, USA). The cannula was purchased from PlasticsOne (Torrington, USA), implanted into the PFC (AP: + 1.7 mm; ML: ± 0.5 mm; DV: –2.2 mm from the bregma; 0° angle), and secured with poly-F dental cement (Dentsply, York, USA) and a screw to the skull (PlasticsOne, Torrington, USA). Each mouse received bilateral mPFC infusions of BDNF (0.25 µg/0.5 µl/side, 248-BDB, R & D Systems, Minnesota, USA) dissolved in 10% DMSO (vol/vol) in saline (Sigma, St. Louis, USA) weekly over the 4 weeks of receiving CUS[4]. ANA-12 (1.0 µg/0.5 µl/side, SML0209, Sigma, St. Louis, USA) was dissolved in 10% DMSO (vol/vol) in saline at a constant rate of 0.1 µl/min with a micro-infusion pump (Legato 200) once every 3 days to block the BDNF receptor[5]. The injectates were administered via a 33-gauge stainless steel internal cannula (2.0 mm C235G-1.5/SPC guide, with a 0.5 mm projection into the mPFC). Recovery from surgery was monitored for a minimum of 3 days post-surgery.

Optogenetic manipulation

In vivo optogenetic manipulation of the mPFC was conducted for 30 min during the SPT after CUS. For photoactivation, custom patch cords with ceramic sleeves (Precision Fiber Products) were attached to the implanted optic fibers and connected to a 473 nm blue laser diode (OEM Laser Systems, Midvale, USA) and a stimulator to generate blue light pulses (lights on, five pulses at 20 Hz, 40 ms pulse durations, every 10 s)[6].

RNA extraction and quantitative real-time PCR (qRT-PCR)

Mouse brains were collected on ice 4 days after the last CUS (1 day after the final SPT). mPFC dissections were obtained using a 14-gauge needle punch (BP-10F, Kai Medical) and promptly frozen at −80°C until RNA extraction.

RNA was isolated with the RNeasy Micro kit (74004, QIAGEN, Hilden, Germany) for qRT-PCR and RNA-sequencing experiments. Subsequently, some RNA samples were sent to Macrogen Inc. (Seoul, Korea) to create a library for RNA-sequencing.

For qRT-PCR experiments, we prepared cDNA using iScript (1708891, Bio-Rad, Hercules, USA). The purity and concentration of RNA and cDNA were measured using Tapestation 4200 (Agilent Technologies, Santa Clara, USA), and only those with RIN values of ≥ 8.0 were used. Primers were designed to amplify the regions of 100–250 base pairs located within the genes: *Syt4*, *F*: TCTTAAAAGCGCGGCACCRA, *R*: ACCAACCGTCCAATCACCTC; and *Gapdh*, *F*: AACTTTGGCATTGTGGAAGG, *R*: ACACATTGGGGGTAGGAAC. SYBR Green qRT-PCR was run in triplicate using a Light Cycler 480II (Roche, Basel, Switzerland) and analyzed using the $\Delta\Delta C_t$ method as previously described, with *Gapdh* as a normalization control[3].

Tamoxifen injection and immunohistochemistry

In Fos-TRAP mice, the experimental schedule of tamoxifen injection was conducted by referring to previously well-established papers[7]. Briefly, tamoxifen (T5648-5G, Sigma-Aldrich, St. Louis, USA) was dissolved in corn oil (C8267-500ML, Sigma-Aldrich, St. Louis, USA) (6 mg/300 μl) and injected into each mouse at a 7.5 μl/g ratio 1 day before the experiment. One week after tamoxifen injection, all mice were anesthetized with CO_2 gas and perfused with 1X PBS and 4% PFA for immunofluorescence investigation. Subsequently, the brain was extracted and post-fixation was performed using 4% PFA, followed by incubation overnight at 4°C. The next day, the brains were washed three times with 1X PBS for 5 min each, before placing in a 30% sucrose solution formulated with 1X PBS and kept at 4°C until frozen sectioning. For immunohistochemistry, the brain was sliced into 50-µm-thick sections at –20°C using a cryostat (Leica, Wetzlar, Germany). Subsequently, the brain slices were washed three times with 1X PBS, for 5 min each, before conducting a blocking step using 4% normal donkey serum (NDS) with 0.3% Triton X-100 in PBS for 1 h. Following blocking, the brain slices were subjected to overnight incubation with the SYT4 antibody (105 043, Synaptic Systems, Göttingen, Germany), at a 1:200 antibody-to-solution ratio. After a 16-h incubation, the slices were washed three times with 1X PBS. Following primary antibody incubation, the sections were incubated with a secondary antibody at a ratio of 1:1000. Post-secondary antibody incubation, the slices underwent further washes with 1X PBS with 0.1% Triton X-100, before counterstaining and mounting the tissues with Hard-set Antifade Mounting Medium containing DAPI (H-1500, Vector Laboratories, Burlingame, USA). Images were taken with a Nikon A1R-MP and Leica STELLARIS 8 confocal microscope. The number of immunopositively cells inside a region of interest (ROI) at each brain region (Figs. 2b and 4c) were counted automatically under the same intensity and threshold in each image and normalized by the number of $DAPI^+$ cells to eliminate between-sample variance[6].

Western blot analysis

Western blot analysis was performed as previously described[6, 8]. Briefly, proteins were extracted from mPFC tissues using RIPA lysis buffer (89900, Thermo Scientific, Massachusetts, USA) including a protease inhibitor cocktail (11697498001, Roche, Basel, Switzerland). The extracted protein was measured using the bicinchoninic acid protein assay kit (BCA1-1KT, Thermo Scientific, Massachusetts, USA). The protein (30 μg) was subjected to SDS-PAGE, the protein was transferred to a 0.2-μm polyvinylidene fluoride (PVDF) membrane (#1620177, Bio-Rad, California, USA), and the membrane were fixed with 2.5% glutaraldehyde (G6403, Sigma) in 1X PBS pH 7.4 for 30 min to detected proBDNF[89]. All membranes were blocked with 3% BSA in 1X TBST (#1706435, Bio-Rad). BDNF, TrKB, Phospho-TrkB, and B-actin proteins were quantified using BDNF (1:2000, ab108319, Abcam, Cambridge, UK), TrkB (1:1000, ab18987, Abcam, Cambridge, UK), Phospho-TrkB (1:500, ABN1381, Millipore, Massachusetts, USA), and B-actin (1:5000, SC-47778, Santa Cruz Biotechnology, Dallas, USA) antibodies, respectively. To measure mature BDNF, proBDNF, P-trkb, and trkb in the same membrane, we removed the antibody using stripping buffer (21059, ThermoFisher, Massachusetts, USA) for 30 min and performed reblocking again. Striping buffer and antibody washing was performed three times for 10 min with 1X TBST, and signals were detected using the LAS-4000 system (Fuji, Tokyo, Japan) with a high-sensitive immobilon western chemiluminescent HRP substrate (WBKLS0500, Millipore, Massachusetts, USA). Western blots were quantified using Image J.

Dopamine ELISA

A dopamine ELISA was conducted to investigate changes in dopamine levels in relation to *Syt4* virus expression. The ENZ-KIT188-0001 dopamine ELISA kit (Enzo Life Sciences in Lausen, Switzerland) was employed for this purpose. Briefly, the conditioned medium of each

sample was added to separate wells, followed by the addition of an equivalent volume of biotin detection antibody. The plates were then incubated at 37°C for 45 min and subsequently washed 3 times. Subsequently, a horseradish peroxidase streptavidin conjugate working solution was introduced and allowed to incubate at 37°C for 30 min. After the incubation period, the samples underwent another round of washing, followed by an incubation with tetramethylbenzidine substrate for 15 min at 37°C in room light. The reaction was halted by the addition of a stop solution. The resulting absorbance, represented as optical density, was measured at 450 nm using a microplate reader.

Clustering analysis

The Waikato Environment for Knowledge Analysis (WEKA) program, the most popular data mining package in the world (version 3. 8. 4), was used to perform clustering analysis. After CUS, expectation maximization (EM), one of the *K*-means cluster algorithms provided by WEKA, was used to classify clusters using *K*-means clustering. The detailed process was introduced in a previous study[10].

PCA analysis

We performed PCA biplot analysis to visualize the relationship between “variables” and “observations” using GraphPad Prism 9 (GraphPad Software, Inc., La Jolla, CA, USA). To create a PCA, we chose the following five distinct behaviors identified through CUS: SPT, ST, SND, FST, and EPM. In a biplot, each observation is represented by a point, and each variable is represented by a vector. The direction of the vector indicates the relationship between the variable and principal component, and the length of the vectors indicates the strength of their contribution to each principal component[11]. The distance between the variables on the biplot

represents the degree of correlation between them, with closer variables indicating a stronger correlation. The biplot also displays the variance explained by each principal component.

HCR™ RNA-FISH

The HCR™ RNA-FISH technique was employed for RNA-FISH experiments, following established protocols[12]. All mice including layer-specific cre mice were perfused, and their brains were fixed overnight in 4% PFA in 1X PBS. Cryoprotection was achieved using 30% sucrose in 1X PBS. We conducted a modified HCR RNA-FISH procedure based on the manufacturer's guidelines (Molecular Instruments). Briefly, a 60-µm-thick brain slice was affixed to a slide and underwent post-fixation, optional ethanol dehydration, and a mild proteinase K treatment (10 µg/ml, 10 min, 37°C) as pre-treatment steps. Subsequently, the slice was incubated with 0.4 pM HCR RNA-FISH probes (*Syt4*, *Slc17a7*, *Slc32a1*) at 37°C overnight. Following a probe wash with 5X SSCT, the signal was developed and amplified using 60-nM hairpin pairs at room temperature overnight. Following the amplification step, the brain slice underwent a 1.05-h wash with 5X SSCT, including periodic buffer changes. All prepared slides were mounted using Mounting Medium with DAPI (H-1500, Vector Laboratories, Burlingame, USA). All materials for FISH were purchased from Molecular Instruments (https://store.molecularinstruments.com/new-bundle/rna-fish).

RNA Sequencing

CUS, SPT, and mPFC dissection were conducted using the same methods described in RNA extraction and qRT-PCR. Library preparations and RNA sequencing were performed by Macrogen Inc. (Seoul, Korea). Total RNA was isolated and transformed into cDNA libraries using the TruSeq Stranded Total RNA LT Sample Prep Gold kit in accordance with the manufacturer's instructions (Illumina, USA). Before reverse transcription, DNA contamination

was eliminated, and RNA was fragmented randomly. RNA fragments were reverse transcribed to cDNA, and adapters were ligated. After PCR amplification, cDNAs of 200–400 bp were selected and sequenced using a NovaSeq 6000 (Illumina, USA) to generate 101 bp paired-end reads. Preprocessing was performed to eliminate artifacts (low quality, adapter sequence, contaminated DNA, or PCR duplicates) from sequenced raw reads using Trimmomatic (version 0.38). From the ends of the reads, reads with base quality less than 3 and those that did not satisfy the sliding window trim criteria (window size = 4, mean quality = 15) were removed. Next, reads with a minimum length less than 36 were removed. The preprocessed reads were mapped to the reference genome (GRCm38/mm10) using the HISAT2 (Hierarchical Indexing for Spliced Alignment of Transcripts, version 2.1.0) program, which can handle spliced reads through the Bowtie2 aligner, to generate aligned reads (Table S2). StringTie (version 1.3.4d) was used for transcript assembly and quantification. The expression profile was extracted by the Fragments Per Kilobase of transcript per Million mapped reads (FPKM) value. Differentially expressed genes (DEGs) were analyzed after excluding genes with FPKM = 0 and quantile normalization of the log2 (FPKM+1) values. Raw and processed data have been deposited in the Gene Expression Omnibus (GSE226576). Genes with a |fold change value| > 1.3 and $p < 0.05$ by Student's *t*-test in each comparison set (control [CTRL] vs. susceptible [SUS], CTRL vs. resilient [RES], and SUS vs. RES) were considered DEGs. The MDS plot was visualized using the ggpubr package (version 0.4.0), and the gene expression heatmap of DEGs was visualized using the gplots package (version 3.1.1) in R (version 4.0.4). Quantile normalized log2 (FPKM+1) values were used to calculate Z-scores in the heatmaps. The venneuler package (version 1.1.0) in R (version 4.0.4) was used to create a Venn diagram.

Weighted gene co-expression network analysis (WGCNA)

The WGCNA package (version 1.70.3) in R (version 4.0.4) was implemented to construct signed co-expression networks using quantile normalized log2 (FPKM+1) values of RNA sequencing. The blockwiseModules function in WGCNA was used to construct a co-expression network and modules. Soft-thresholding power was chosen (power = 17) according to a scale-free topology criterion. We set the minimum module size to 20 genes, the deep split to 2, and the minimum height for merging modules to 0.15. Each module was summarized by an eigengene value, which is the first principal component of the expression matrix. To build exemplary modules, we set a threshold on the module membership measure (also known as module eigengene-based connectivity, kME), which is the correlation between gene expression values and the module eigengene values. Only genes with kME > 0.7 were included in the modules. The module membership was also used to rank genes in the module. The top ten genes in the rank were considered hub genes of the module. Following construction of the gene co-expression network and module, we investigated the relationships between the modules and sample traits by linear regression and Pearson correlation. Among 203 modules, 43 were significantly associated with sample groups (CTRL, RES, and SUS) in linear regression ($p < 0.05$). Module-trait relationships were also calculated using Pearson correlation between module eigengene and traits (CUS or stress-susceptibility) to identify the effects of CUS and stress-susceptibility separately. The *p*-values of module-trait correlations were calculated using the corPvalueStudent function in the WGCNA package. Six modules were selected on the basis of module-trait relationships, module size, and expression patterns. Between the top two modules of the most positively or negatively correlated modules with CUS or stress-susceptibility, the modules with larger sizes were selected (M91, M199, M166, and M203) to investigate the biological functions related to a greater number of genes. M33 (RES-up) and M104 (RES-down) were selected to characterize gene modules that were specifically upregulated or downregulated in RES, and they were the largest modules among the RES-up

and RES down modules, respectively. The gene expression heatmap of each module was visualized in the same manner as the DEG heatmap, and the intramodular gene co-expression network was visualized using Cytoscape (version 3.8.2). The thickness and darkness of edges in the networks represent the correlation of expression values between two genes.

Gene functional enrichment analysis

Gene ontology (GO) and Kyoto Encyclopedia of Genes and Genomes (KEGG) pathway enrichment analyses were performed to explore the functional roles of DEGs from mRNA-seq data and genes in the selected modules using the Database for Annotation, Visualization and Integrated Discovery version 2021 (DAVID; http://david.ncifcrf.gov/). The enrichment results were visualized using the ggplot2 package (version 3.3.3) of the R program (version 4.0.4). Statistically significant categories were selected (p-value < 0.05).

Cell-type enrichment analysis

The cell type marker gene lists were composed based on single-cell RNA sequencing data from the Allen brain map database. Genes with a mean expression value in a specific cell cluster that was more than four times the mean expression value in all cell clusters were defined as a cell type marker gene of the specific cell cluster. Cell-type enrichment analysis on M166 was performed using these cell-type marker genes and Fisher's exact test. The -log10 (Benjamini-Hochberg adjusted p-values) of Fisher's exact test were calculated for M166.

Data and statistical analysis

Except for the two-bottle SPT, NSF, home cage feeding, and FST, all behavioral tests (e.g., three-chamber ST, SND test, and EPM test) were recorded using the automated and unbiased SMART3.0 video tracking system (Panlab). All values are expressed as the mean ± standard

error of the mean (SEM). All statistical analyses, including PCA, were performed using GraphPad Prism 9 (GraphPad Software), Origin Pro 8 (OriginLab), and R (version 4.0.4). The sample size, normality, and homoscedasticity of the distributions were used to examine the statistic sample values. Shapiro–Wilk test and Bartlett's test (or F-test) were used for normality and tests of equal variances. The specific statistical tests and their results are described in the figure legends. Grubb's test, an outlier analysis enabled by GraphPad Prism 9, was used to remove outliers from the data.

Supplementary Figures

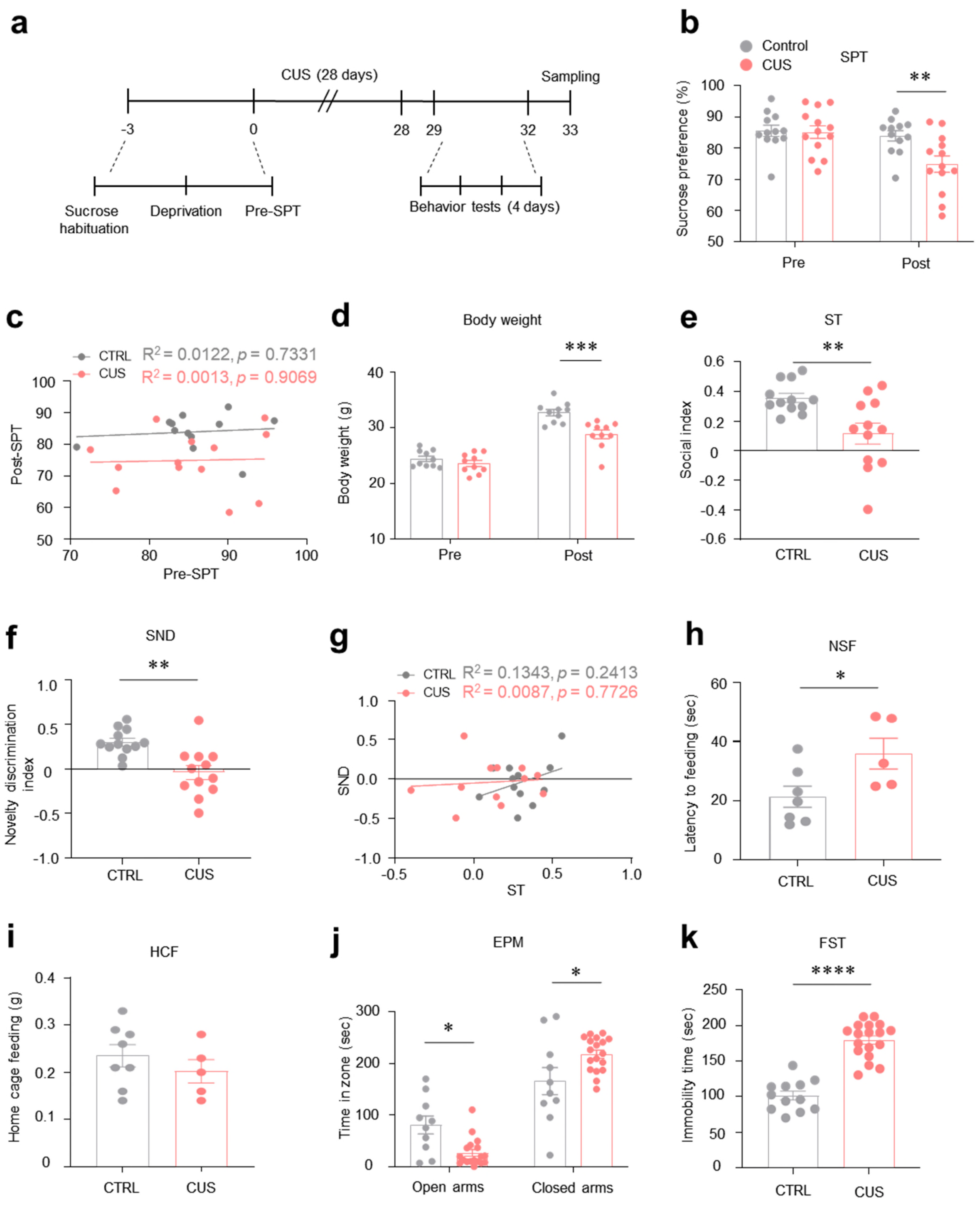

Supplementary Fig. 1. Chronic unpredictable stress (CUS) mediated depressive-like behaviors. (a) Experimental procedures for CUS. (b) Sucrose preference in male mice before and after CUS (two-way ANOVA with Bonferroni's multiple comparisons post hoc test, stress effect: $F_{1, 46} = 5.111$, $p = 0.0285$; time effect (pre vs. post): $F_{1, 46} = 8.038$, $p = 0.0068$; interaction: $F_{1, 46} = 4.281$, $p = 0.0442$, $n = 12, 13$). (c) One-way ANCOVA based on the pre-sucrose preference test (SPT) ($F_{1, 21} = 0.01185$, $p = 0.9144$, $n = 12, 13$). (d) Bar graph showing a comparison of the body weight before and after CUS (two-way ANOVA with Bonferroni's multiple comparisons post hoc test, stress effect: F1, 36 = 15.52, $p < 0.0004$; time effect (pre vs. post): F1, 36 = 125.9, $p < 0.0001$; interaction: F1, 36 = 6.408, $p = 0.0159$, $n = 10, 10$). (e) CUS mice exhibited reduced social interaction in the sociability test (unpaired t-test with Welch's correction, $t_{14.89} = 3.147$, $p = 0.0067$, $n = 12, 12$). (f) CUS reduced the social novelty discrimination compared to control (CTRL) animals (unpaired t-test, $t_{22} = 3.789$, $p = 0.0010$, $n = 12, 12$). (g) One-way ANCOVA based on ST ($F_{1, 20} = 0.7579$, $p = 0.3943$, $n = 12$). (h) CUS significantly increased the latency to eat chow centered in an unfamiliar space in the novelty suppressed feeding (NSF) test (unpaired t-test, $t_{10} = 2.386 = 10$, $p < 0.0001$, $n = 7, 5$). (i) Amount of home cage feeding after the NSF (unpaired t-test, $t_{11} = 0.9250$, $p = 0.3748$, $n = 7, 5$). (j) The CUS group spent less time in the open arms and more time in the closed arms in the elevated plus maze (EPM) compared to the CTRL group (two-way ANOVA with Bonferroni's multiple comparisons post hoc test, stress effect: F1, 52 = 0.005120, $p = 0.9432$; maze effect (open vs. closed arms): F1, 52 = 105.6, $p < 0.0001$; interaction: F1, 52 = 15.59, $p = 0.0002$, $n = 10, 18$). (k) CUS animals struggled for less time in the FST compared to the CTRL animals (unpaired t-test, $t_{28} = 8.918$, $p < 0.0001$, $n = 12, 18$). $^{*}p < 0.05$, $^{**}p < 0.01$, $^{***}p < 0.001$, $^{****}p < 0.0001$. The bar graphs show the mean ± SEM.

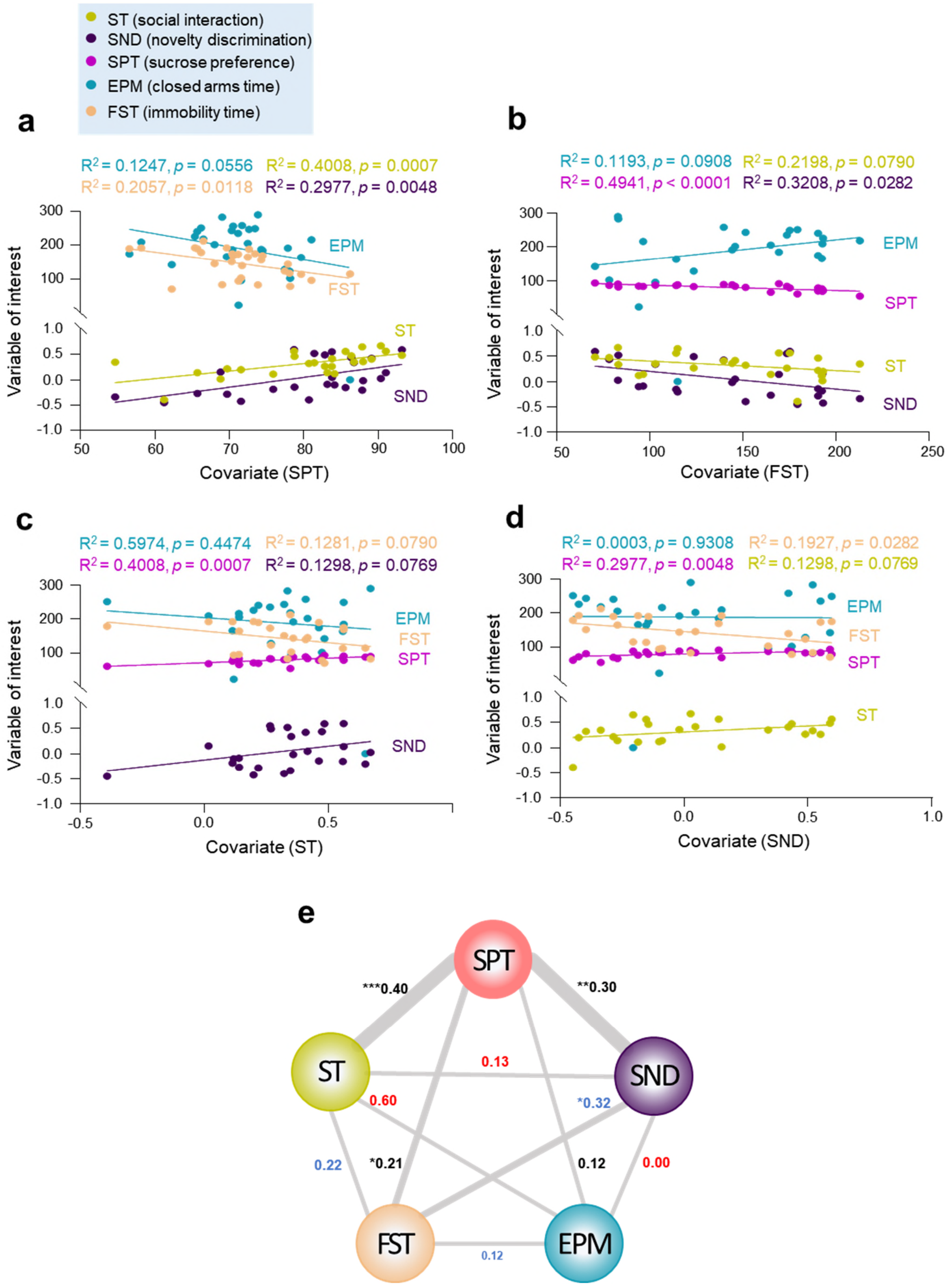
ST (social interaction)
SND (novelty discrimination)
SPT (sucrose preference)
EPM (closed arms time)
FST (immobility time)
a
R2 = 0.1247, p = 0.0556
R2 = 0.4008, p = 0.0007
R2 = 0.2057, p = 0.0118
R2 = 0.2977, p = 0.0048
Variable of interest
Covariate (SPT)
EPM
FST
ST
SND
b
R2 = 0.1193, p = 0.0908
R2 = 0.2198, p = 0.0790
R2 = 0.4941, p < 0.0001
R2 = 0.3208, p = 0.0282
Variable of interest
Covariate (FST)
EPM
SPT
ST
SND
c
R2 = 0.5974, p = 0.4474
R2 = 0.1281, p = 0.0790
R2 = 0.4008, p = 0.0007
R2 = 0.1298, p = 0.0769
Variable of interest
Covariate (ST)
EPM
FST
SPT
SND
d
R2 = 0.0003, p = 0.9308
R2 = 0.1927, p = 0.0282
R2 = 0.2977, p = 0.0048
R2 = 0.1298, p = 0.0769
Variable of interest
Covariate (SND)
EPM
FST
SPT
ST
e
SPT
ST
SND
FST
EPM
***0.40
**0.30
0.13
0.60
*0.32
0.22
*0.21
0.12
0.00
0.12

Supplementary Fig. 2. Analysis of covariance between multiple behavioral analyses based on sucrose preference after CUS. (a) One-way ANCOVA based on the sucrose preference ($F_{3, 102}$ = 3.897, p = 0.0111, n = 23, 31). (b) One-way ANCOVA based on the FST immobility time ($F_{3, 92}$ = 3.884, p = 0.0116, n = 12, 13). (c) One-way ANCOVA based on the ST ($F_{3, 92}$ = 1.360, p = 0.2599, n = 12, 13). (d) One-way ANCOVA based on the SND ($F_{3, 92}$ = 1.497, p = 0.2205, n = 12, 13). (e) Correlations between multiple depressive-like behaviors. Values in black shown in the plot correspond to R^2 from ANCOVA between SPT and other depressive-like behaviors. Values in blue are from the FST or EPM and other depressive-like behaviors in the CTRL and CUS datasets of Supplementary Fig. 1. Values in red are from the ST or SND test in the CTRL and CUS datasets of Supplementary Fig. 1. The thickness of the lines indicates the strength of the individual correlations. $^{*}p < 0.05$, $^{**}p < 0.01$, $^{***}p < 0.001$.

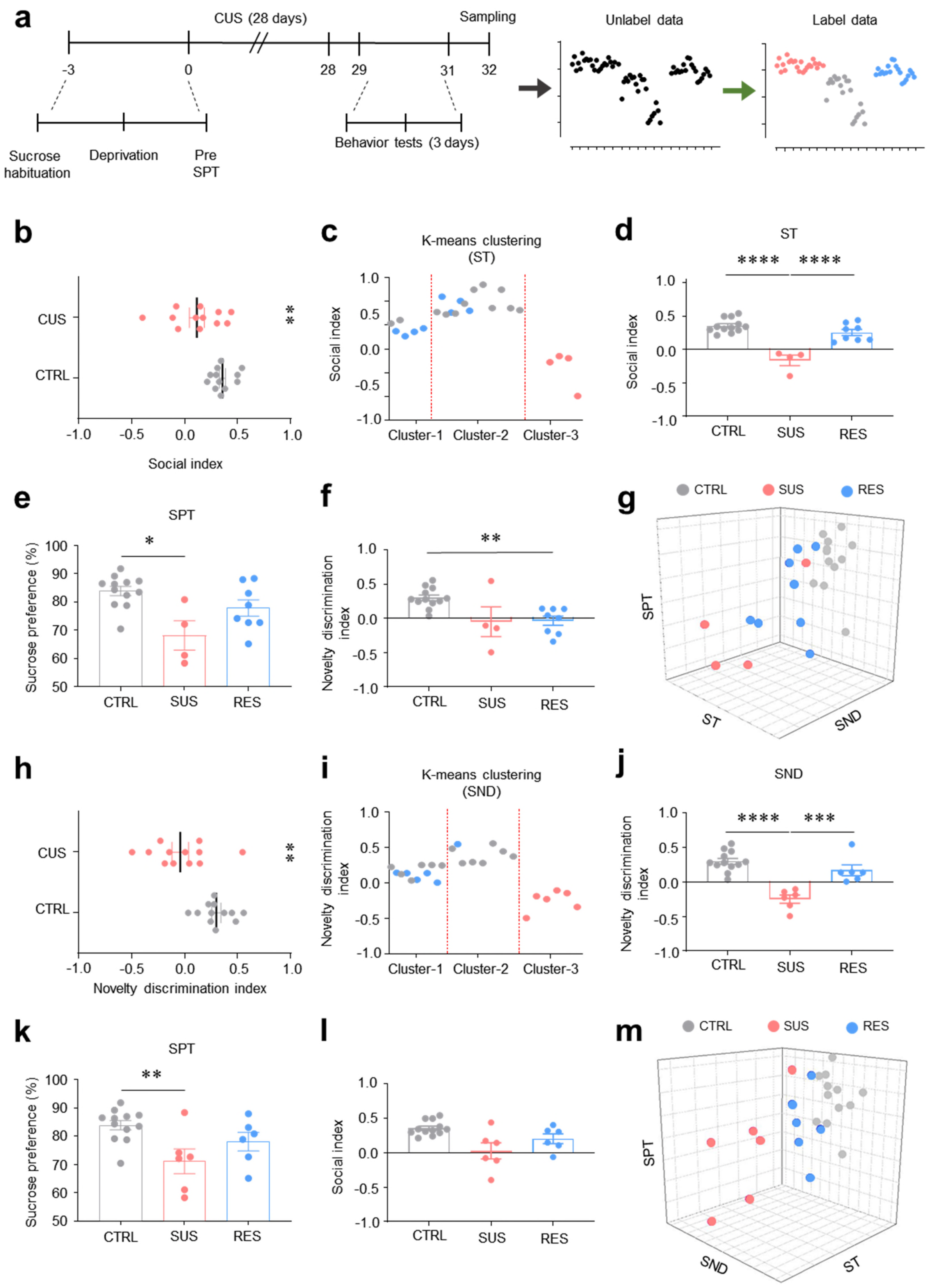
a
CUS (28 days)
Sampling
-3
0
28
29
31
32
Sucrose habituation
Deprivation
Pre SPT
Behavior tests (3 days)
Unlabel data
Label data
b
CUS
CTRL
Social index
c
K-means clustering (ST)
Social index
Cluster-1
Cluster-2
Cluster-3
d
ST
CTRL
SUS
RES
e
SPT
Sucrose preference (%)
f
Novelty discrimination index
g
SPT
ST
SND
h
Novelty discrimination index
i
K-means clustering (SND)
j
SND
k
l
m

Supplementary Fig. 3. Separation of subpopulations based on social anhedonia and novelty social recognition after CUS. (a) Experimental procedures for CUS and *K*-means clustering. (b) CUS significantly increased social anhedonia (unpaired t-test; $t_{22} = 3.147$, $p = 0.0047$, $n = 12$). (c) *K*-means clustering after CUS based on ST. (d) ST-susceptible (SUS) mice showed increased anhedonia behavior compared to CTRL and ST-RES animals (one-way ANOVA with Tukey's multiple comparisons post hoc test, $F_{2, 21} = 27.55$, $p < 0.0001$, $n = 12$, 4, 8). (e) ST-SUS animals showed decreased sucrose preference compared to CTRL animals, but no significant difference compared to RES animals (one-way ANOVA with Tukey's multiple comparisons post hoc test, $F_{2, 21} = 7.055$, $p = 0.0045$, $n = 12$, 4, 8). (f) The results showed no significant difference between ST-SUS and CTRL, although ST-RES mice exhibited decreased novelty discrimination compared to CTRL mice (one-way ANOVA with Welch's test, $W_{2.000, 6.692} = 8.656$, $p = 0.0139$, $n = 12$, 4, 8). (g) A 3D plot depicting the results from the ST, SND test, and SPT for individual SUS, RES, and CTRL mice in the same cohort. (h) CUS significantly decreased novelty discrimination (unpaired t-test; $t_{22} = 3.789$, $p = 0.0010$, $n = 12$). (i) *K*-means clustering after CUS based on SND. (j) SND-SUS mice showed decreased novelty social recognition compared to CTRL and ST-RES animals (one-way ANOVA with Tukey's multiple comparisons post hoc test, $F_{2, 21} = 24.06$, $p < 0.0001$, $n = 12$, 6, 6). (k) SND-SUS animals showed decreased sucrose preference compared to CTRL, but no significant difference compared to RES animals (one-way ANOVA with Tukey's multiple comparisons post hoc test, $F_{2, 21} = 5.535$, $p = 0.0117$, $n = 12$, 6, 6). (l) When divided by SND, there was no significant difference in social anhedonia among all groups (one-way ANOVA with Welch's test, $W_{2.000, 8.122} = 4.816$, $p = 0.0418$, $n = 12$, 6, 6). (m) A 3D plot depicting the results from the SND test, ST, and SPT for each individual SUS, RES, and CTRL mice in the same cohort. $^{*}p < 0.05$, $^{**}p < 0.01$, $^{***}p < 0.001$, $^{****}p < 0.0001$. The bar graphs show the mean ± SEM.

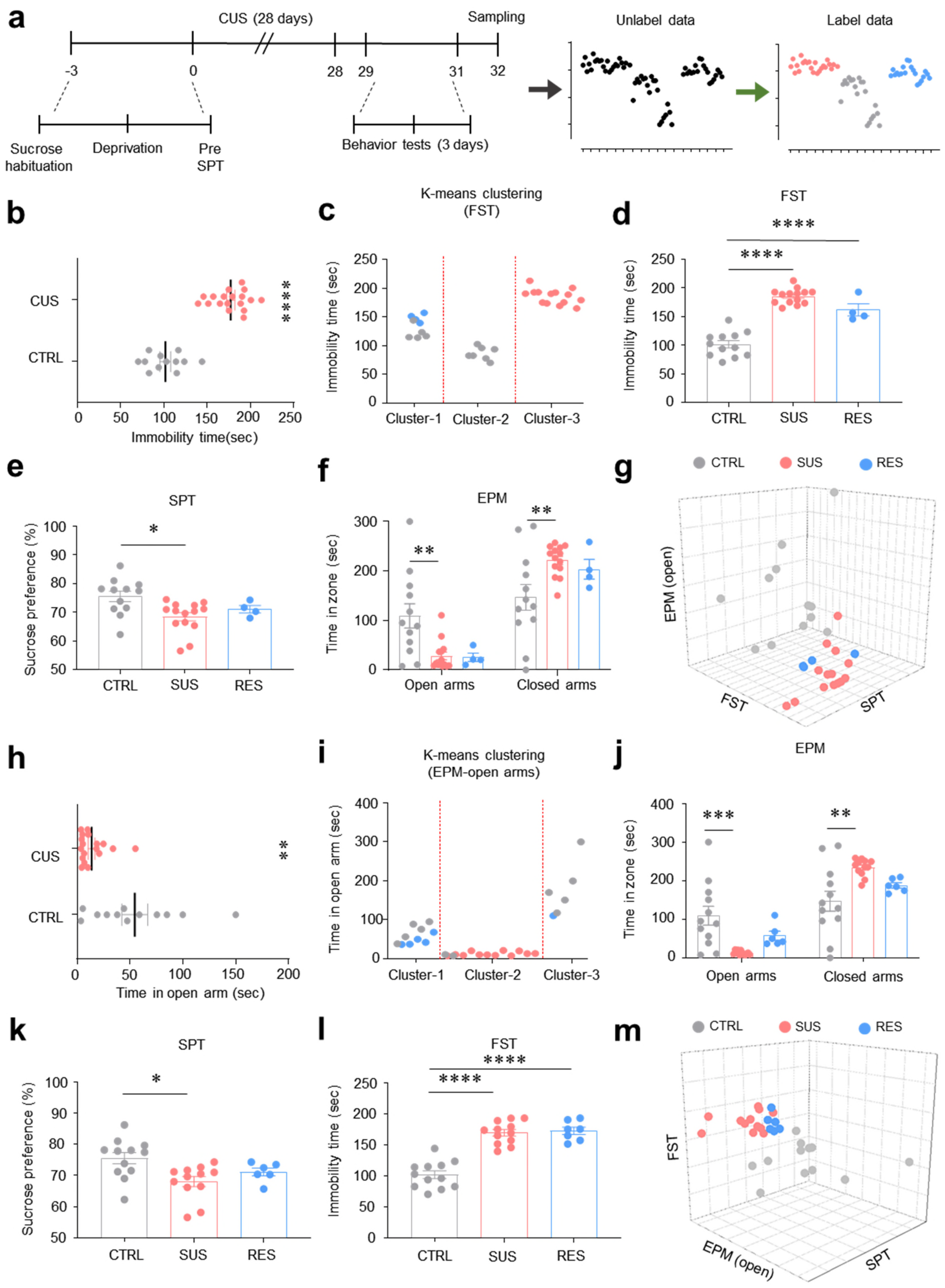
a
CUS (28 days)
Sampling
-3
0
28
29
31
32
Sucrose habituation
Deprivation
Pre SPT
Behavior tests (3 days)
Unlabel data
Label data
b
CUS
CTRL
****
Immobility time(sec)
c
K-means clustering (FST)
Immobility time (sec)
Cluster-1
Cluster-2
Cluster-3
d
FST
****
****
Immobility time (sec)
CTRL
SUS
RES
e
SPT
*
Sucrose preference (%)
CTRL
SUS
RES
f
EPM
**
**
Time in zone (sec)
Open arms
Closed arms
g
CTRL
SUS
RES
EPM (open)
FST
SPT
h
CUS
CTRL
**
Time in open arm (sec)
i
K-means clustering (EPM-open arms)
Time in open arm (sec)
Cluster-1
Cluster-2
Cluster-3
j
EPM
***
**
Time in zone (sec)
Open arms
Closed arms
k
SPT
*
Sucrose preference (%)
CTRL
SUS
RES
l
FST
****
****
Immobility time (sec)
CTRL
SUS
RES
m
CTRL
SUS
RES
FST
EPM (open)
SPT

Supplementary Fig. 4. Separation of subpopulations based on behavioral despair and anxiety-like behavior after CUS. (a) Experimental procedures for CUS and *K*-means clustering. (b) CUS significantly increased despair-like behavior (unpaired t-test; $t_{9.844} = 28$, $p < 0.0001$, $n = 12, 18$). (c) *K*-means clustering after CUS based on the FST. (d) FST-SUS and RES mice showed significantly increased immobility time compared to CTRL animals (one-way ANOVA with Tukey's multiple comparisons post hoc test, $F_{2, 27} = 70.90$, $p < 0.0001$, $n = 12, 14, 4$). (e) Only FST-SUS animals showed decreased sucrose preference compared to CTRL animals (Kruskal–Wallis test with Dunn's multiple comparisons post hoc test, $H = 8.114$, $p = 0.0173$, $n = 12, 14, 4$). (f) Compared to CTRL animals, only FST-SUS animals showed considerably increased anxiety symptoms (two-way ANOVA with Bonferroni's multiple comparisons post hoc test, stress effect: $F_{2, 54} = 0.1472$, $p = 0.8635$; maze effect (open vs. closed arms): $F_{1, 54} = 56.27$, $p < 0.0001$; interaction: $F_{2,54} = 11.42$, $p < 0.0001$, $n = 12, 14, 4$). (g) A 3D plot depicting the results from FST, SPT, and EPM for individual SUS, RES, and CTRL mice in the same cohort. (h) CUS significantly induced anxiety-like behavior (Mann Whitney U-test; $U = 39$, $p = 0.0026$, $n = 12, 18$). (i) *K*-means clustering after CUS based on EPM data. (j) EPM-SUS mice showed increased anxiety-like behavior compared to CTRL mice (two-way ANOVA with Bonferroni's multiple comparisons post hoc test, stress effect: $F_{2, 54} = 0.05$, $p = 0.9513$; maze effect (open vs. closed arms): $F_{1.54} = 68.34$, $p < 0.0001$; interaction: $F_{2,54} = 15.24$, $p < 0.0001$, $n = 12, 12, 6$). (k) Only EPM-SUS animals showed decreased sucrose preference compared to CTRL animals (Kruskal–Wallis test with Dunn's multiple comparisons post hoc test, $H = 8.439$, $p = 0.0147$, $n = 12, 12, 6$). (l) EPM-SUS and RES mice showed significantly increased despair behavior compared to CTRL mice. (one-way ANOVA with Tukey's multiple comparisons post hoc test, $F_{2, 28} = 47.53$, $p < 0.0001$, $n = 12, 12, 6$). (m) A 3D plot depicting the FST, SPT, and EPM results for individual SUS, RES, and

CTRL mice in the same cohort. $^{*}p < 0.05$, $^{**}p < 0.01$, $^{***}p < 0.001$, $^{****}p < 0.0001$. The bar graphs show the mean ± SEM.

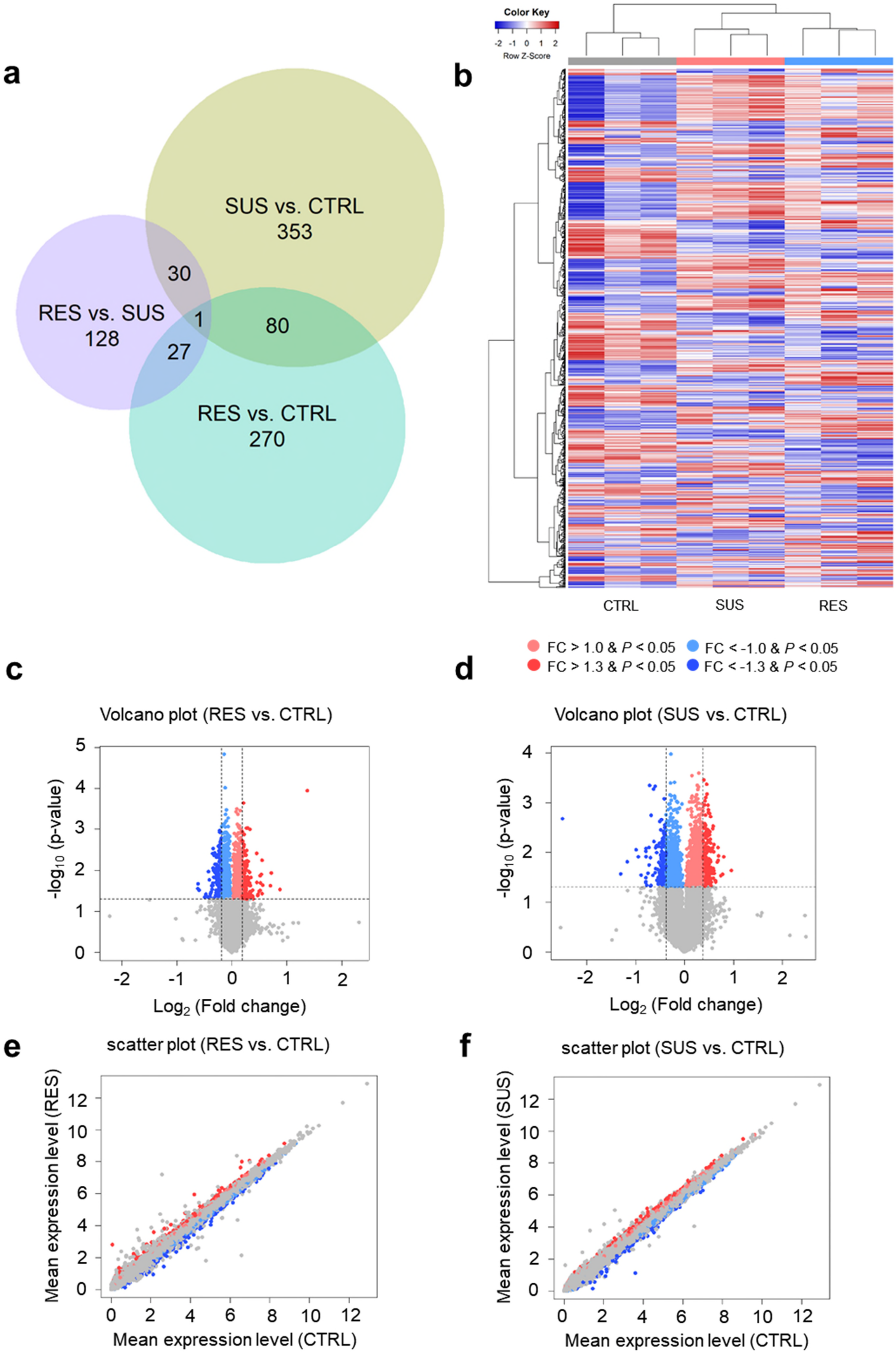
a
SUS vs. CTRL
353
RES vs. SUS
128
30
1
27
80
RES vs. CTRL
270
b
Color Key
Row Z-Score
CTRL
SUS
RES
FC > 1.0 & P < 0.05
FC > 1.3 & P < 0.05
FC < -1.0 & P < 0.05
FC < -1.3 & P < 0.05
c
Volcano plot (RES vs. CTRL)
-log10 (p-value)
Log2 (Fold change)
d
Volcano plot (SUS vs. CTRL)
-log10 (p-value)
Log2 (Fold change)
e
scatter plot (RES vs. CTRL)
Mean expression level (RES)
Mean expression level (CTRL)
f
scatter plot (SUS vs. CTRL)
Mean expression level (SUS)
Mean expression level (CTRL)

Supplementary Fig. 5. Gene expression differs significantly between groups divided by sucrose preference. (a) Distribution of the number of differentially expressed genes (DEGs) between groups. (b) The expression patterns of DEGs between groups were visualized. (c and d) Volcano plots displaying DEGs between (c) RES and CTRL animals and between (d) SUS and CTRL animals. Genes that were considerably more or less expressed in the former groups are shown by red and blue data points, respectively. Gray points represent genes that were not significantly differentially expressed in either group. (e and f) Scatter plot showing the DEGs between (e) RES and CTRL animals and between (f) SUS and CTRL animals. Genes that are considerably more or less expressed in the former groups are indicated by red and blue data points, respectively. Gray points represent genes that are not significantly differentially expressed in either group.

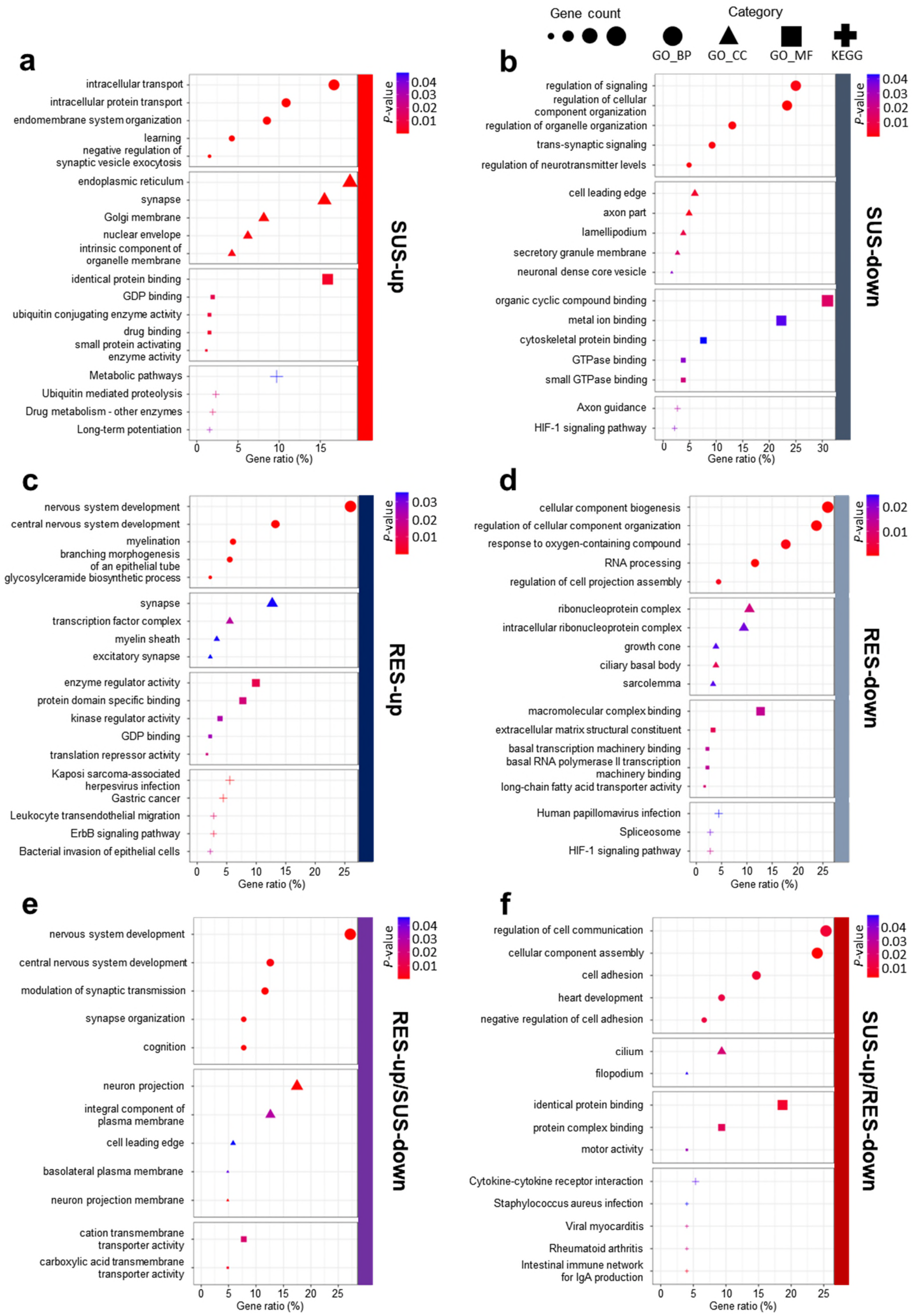

Gene count
Category
GO_BP
GO_CC
GO_MF
KEGG
a
intracellular transport
intracellular protein transport
endomembrane system organization
learning
negative regulation of synaptic vesicle exocytosis
endoplasmic reticulum
synapse
Golgi membrane
nuclear envelope
intrinsic component of organelle membrane
identical protein binding
GDP binding
ubiquitin conjugating enzyme activity
drug binding
small protein activating enzyme activity
Metabolic pathways
Ubiquitin mediated proteolysis
Drug metabolism - other enzymes
Long-term potentiation
Gene ratio (%)
P-value
SUS-up
b
regulation of signaling
regulation of cellular component organization
regulation of organelle organization
trans-synaptic signaling
regulation of neurotransmitter levels
cell leading edge
axon part
lamellipodium
secretory granule membrane
neuronal dense core vesicle
organic cyclic compound binding
metal ion binding
cytoskeletal protein binding
GTPase binding
small GTPase binding
Axon guidance
HIF-1 signaling pathway
Gene ratio (%)
SUS-down
c
nervous system development
central nervous system development
myelination
branching morphogenesis of an epithelial tube
glycosylceramide biosynthetic process
synapse
transcription factor complex
myelin sheath
excitatory synapse
enzyme regulator activity
protein domain specific binding
kinase regulator activity
GDP binding
translation repressor activity
Kaposi sarcoma-associated herpesvirus infection
Gastric cancer
Leukocyte transendothelial migration
ErbB signaling pathway
Bacterial invasion of epithelial cells
Gene ratio (%)
RES-up
d
cellular component biogenesis
regulation of cellular component organization
response to oxygen-containing compound
RNA processing
regulation of cell projection assembly
ribonucleoprotein complex
intracellular ribonucleoprotein complex
growth cone
ciliary basal body
sarcolemma
macromolecular complex binding
extracellular matrix structural constituent
basal transcription machinery binding
basal RNA polymerase II transcription machinery binding
long-chain fatty acid transporter activity
Human papillomavirus infection
Spliceosome
HIF-1 signaling pathway
Gene ratio (%)
RES-down
e
nervous system development
central nervous system development
modulation of synaptic transmission
synapse organization
cognition
neuron projection
integral component of plasma membrane
cell leading edge
basolateral plasma membrane
neuron projection membrane
cation transmembrane transporter activity
carboxylic acid transmembrane transporter activity
Gene ratio (%)
RES-up/SUS-down
f
regulation of cell communication
cellular component assembly
cell adhesion
heart development
negative regulation of cell adhesion
cilium
filopodium
identical protein binding
protein complex binding
motor activity
Cytokine-cytokine receptor interaction
Staphylococcus aureus infection
Viral myocarditis
Rheumatoid arthritis
Intestinal immune network for IgA production
Gene ratio (%)
SUS-up/RES-down

Supplementary Fig. 6. Comparison of the biological functions of DEGs between groups using GO and KEGG enrichment analyses. (a–f) GO and KEGG enrichment analysis results of (a) SUS-up, (b) SUS-down, (c) RES-up, (d) RES-down, (e) RES-up/SUS-down, and (f) SUS-up/RES-down DEGs imply the biological role of DEGs in each comparison.

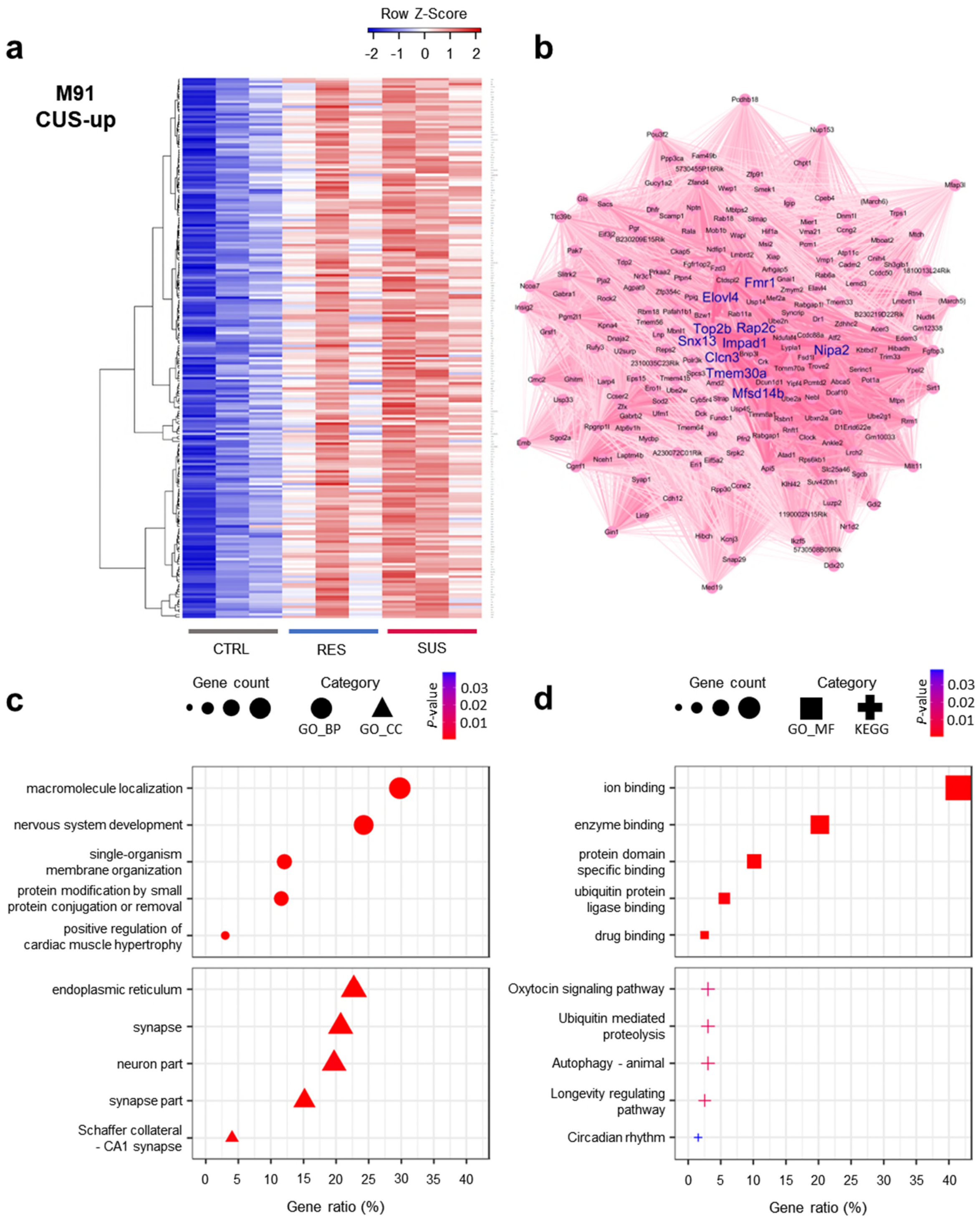
a
M91
CUS-up
Row Z-Score
-2 -1 0 1 2
CTRL
RES
SUS
b
Fmr1
Elovl4
Top2b
Rap2c
Snx13
Impad1
Clcn3
Nipa2
Tmem30a
Mfsd14b
c
Gene count
Category
GO_BP
GO_CC
P-value
0.03
0.02
0.01
macromolecule localization
nervous system development
single-organism membrane organization
protein modification by small protein conjugation or removal
positive regulation of cardiac muscle hypertrophy
endoplasmic reticulum
synapse
neuron part
synapse part
Schaffer collateral - CA1 synapse
0 5 10 15 20 25 30 35 40
Gene ratio (%)
d
Gene count
Category
GO_MF
KEGG
P-value
0.03
0.02
0.01
ion binding
enzyme binding
protein domain specific binding
ubiquitin protein ligase binding
drug binding
Oxytocin signaling pathway
Ubiquitin mediated proteolysis
Autophagy - animal
Longevity regulating pathway
Circadian rhythm
0 5 10 15 20 25 30 35 40
Gene ratio (%)

Supplementary Fig. 7. Characterization of the CUS-up module (M91). (a) Module genes were upregulated in RES and SUS groups. (b) Visualization of the intramodular co-expression network of a gene module specifically overexpressed in the CUS group. (c) GO enrichment analysis of biological processes and cellular components of the CUS-up module. (d) Enrichment analysis of GO molecular function and KEGG analysis of CUS-up module genes.

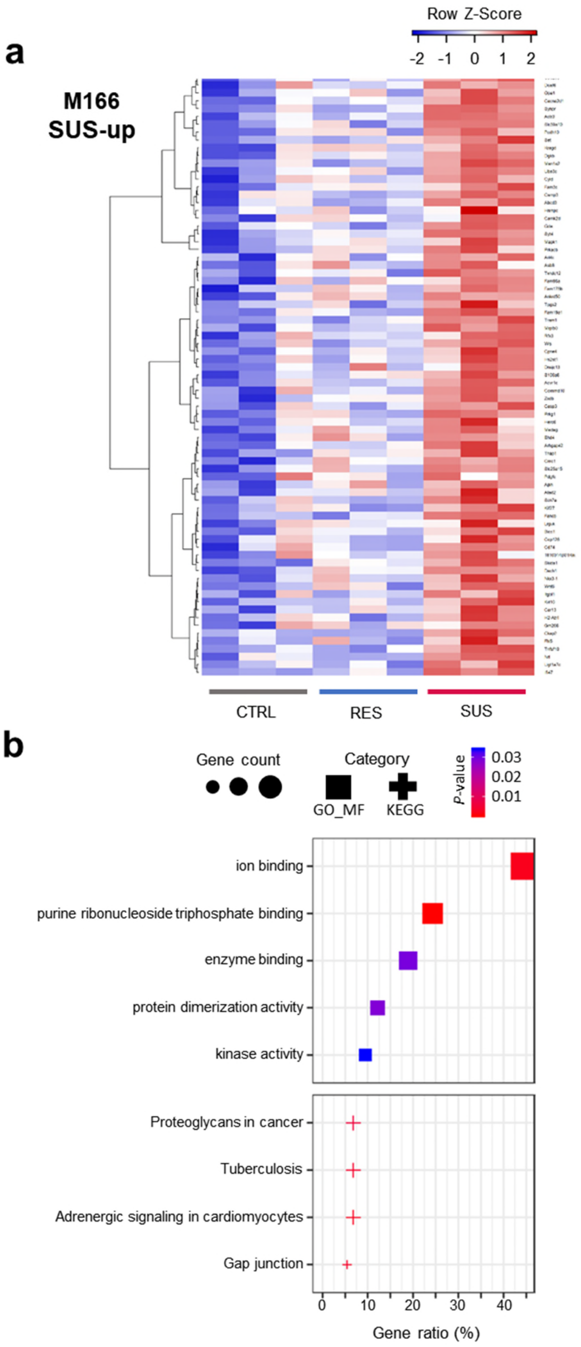
a
M166
SUS-up
Row Z-Score
-2 -1 0 1 2
CTRL
RES
SUS
b
Gene count
Category
GO_MF
KEGG
P-value
0.03
0.02
0.01
ion binding
purine ribonucleoside triphosphate binding
enzyme binding
protein dimerization activity
kinase activity
Proteoglycans in cancer
Tuberculosis
Adrenergic signaling in cardiomyocytes
Gap junction
0
10
20
30
40
Gene ratio (%)

Supplementary Fig. 8. Characterization of the SUS-up module (M166). (a) Module genes were specifically upregulated in the SUS group. (b) Enrichment analysis of GO molecular function and KEGG analysis of SUS-up module genes.

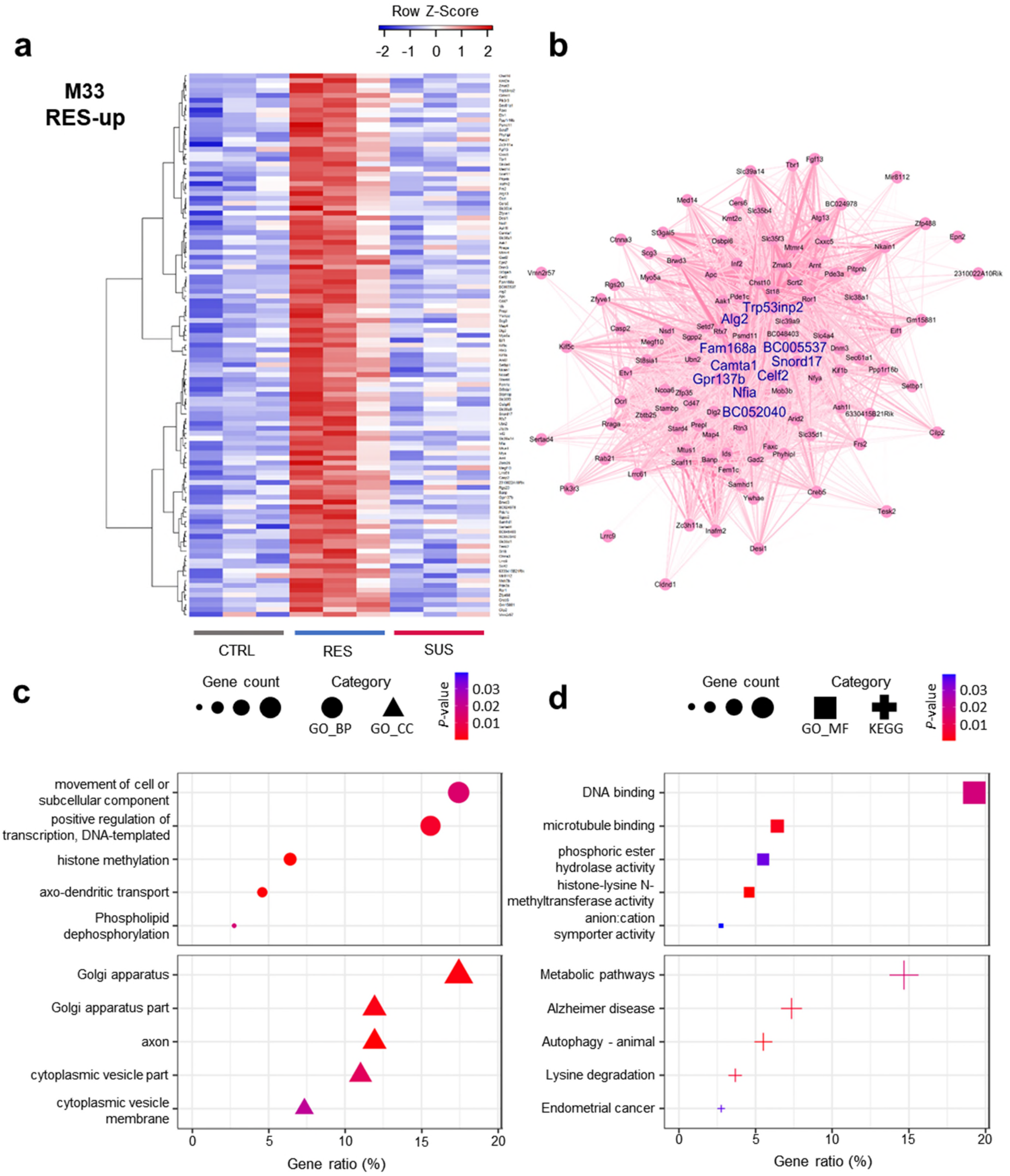
a
b
M33
RES-up
Row Z-Score
-2 -1 0 1 2
CTRL
RES
SUS
Trp53inp2
Alg2
Fam168a
BC005537
Snord17
Camta1
Celf2
Gpr137b
Nfia
BC052040
c
Gene count
Category
GO_BP
GO_CC
P-value
0.03
0.02
0.01
movement of cell or subcellular component
positive regulation of transcription, DNA-templated
histone methylation
axo-dendritic transport
Phospholipid dephosphorylation
Golgi apparatus
Golgi apparatus part
axon
cytoplasmic vesicle part
cytoplasmic vesicle membrane
0 5 10 15 20
Gene ratio (%)
d
Gene count
Category
GO_MF
KEGG
P-value
0.03
0.02
0.01
DNA binding
microtubule binding
phosphoric ester hydrolase activity
histone-lysine N-methyltransferase activity
anion:cation symporter activity
Metabolic pathways
Alzheimer disease
Autophagy - animal
Lysine degradation
Endometrial cancer
0 5 10 15 20
Gene ratio (%)

Supplementary Fig. 9. Characterization of the RES-up module (M33). (a) Module genes were specifically upregulated in the RES group. (b) Visualization of the intramodular co-expression network of a gene module that was specifically over-expressed in the RES group. (c) GO enrichment analysis of the biological processes and cellular components of the RES-up module. (d) Enrichment analysis of GO molecular function and KEGG analysis of RES-up module genes.

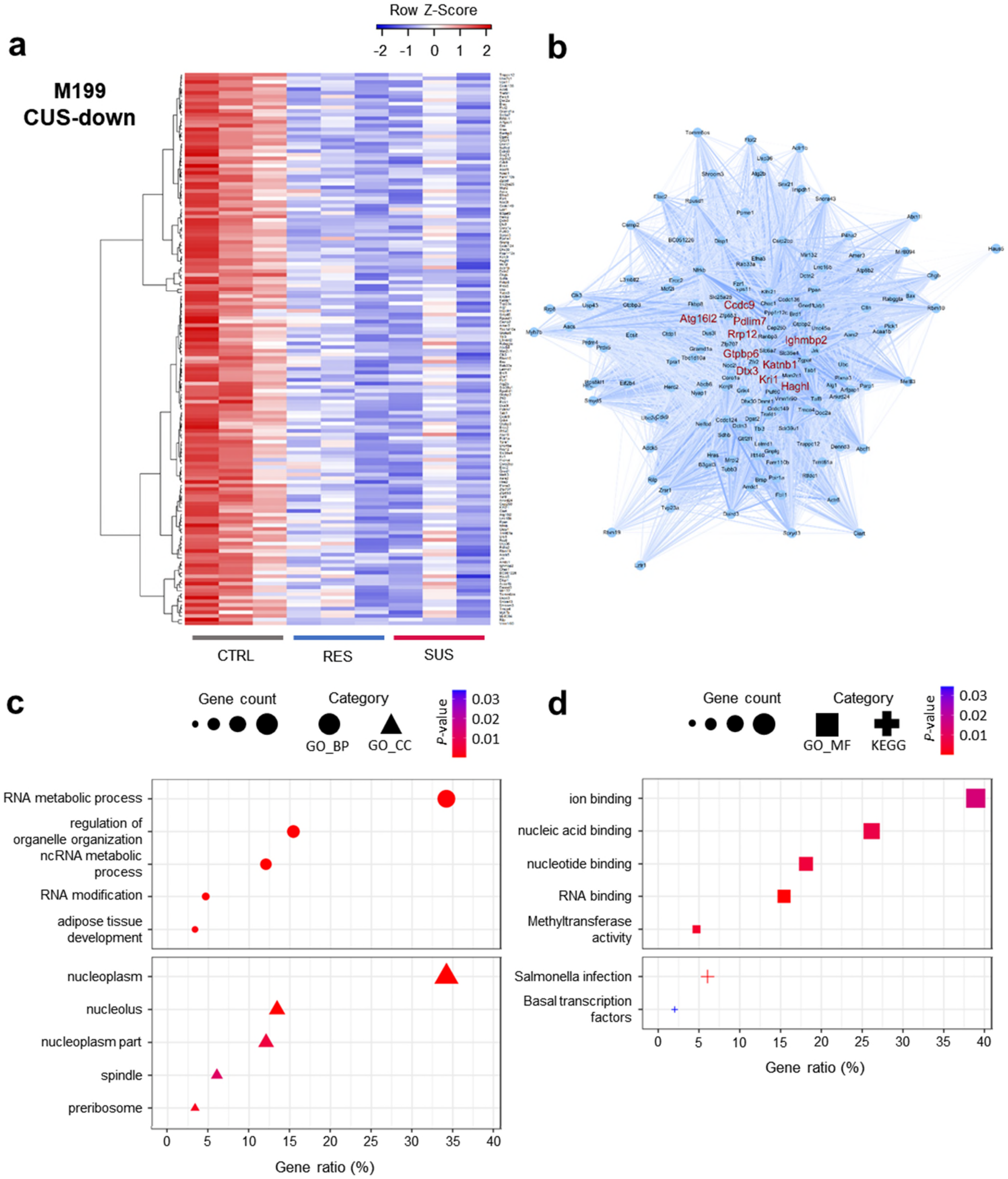
a
b
c
d
Row Z-Score
-2 -1 0 1 2
M199
CUS-down
CTRL
RES
SUS
Ccdc9
Atg16l2
Pdlim7
Rrp12
Ighmbp2
Gtpbp6
Katnb1
Dtx3
Kri1
Haghl
Gene count
Category
GO_BP
GO_CC
P-value
0.03
0.02
0.01
RNA metabolic process
regulation of organelle organization
ncRNA metabolic process
RNA modification
adipose tissue development
nucleoplasm
nucleolus
nucleoplasm part
spindle
preribosome
0 5 10 15 20 25 30 35 40
Gene ratio (%)
Gene count
Category
GO_MF
KEGG
P-value
0.03
0.02
0.01
ion binding
nucleic acid binding
nucleotide binding
RNA binding
Methyltransferase activity
Salmonella infection
Basal transcription factors
0 5 10 15 20 25 30 35 40
Gene ratio (%)

Supplementary Fig. 10. Characterization of the CUS-down module (M199). (a) Module genes were downregulated in the RES and SUS groups. (b) Visualization of the intramodular co-expression network of a gene module specifically repressed in the CUS group. (c) GO enrichment analysis of the biological processes and cellular components of the CUS-down module. (d) Enrichment analysis of GO molecular function and KEGG analysis of CUS-down module genes.

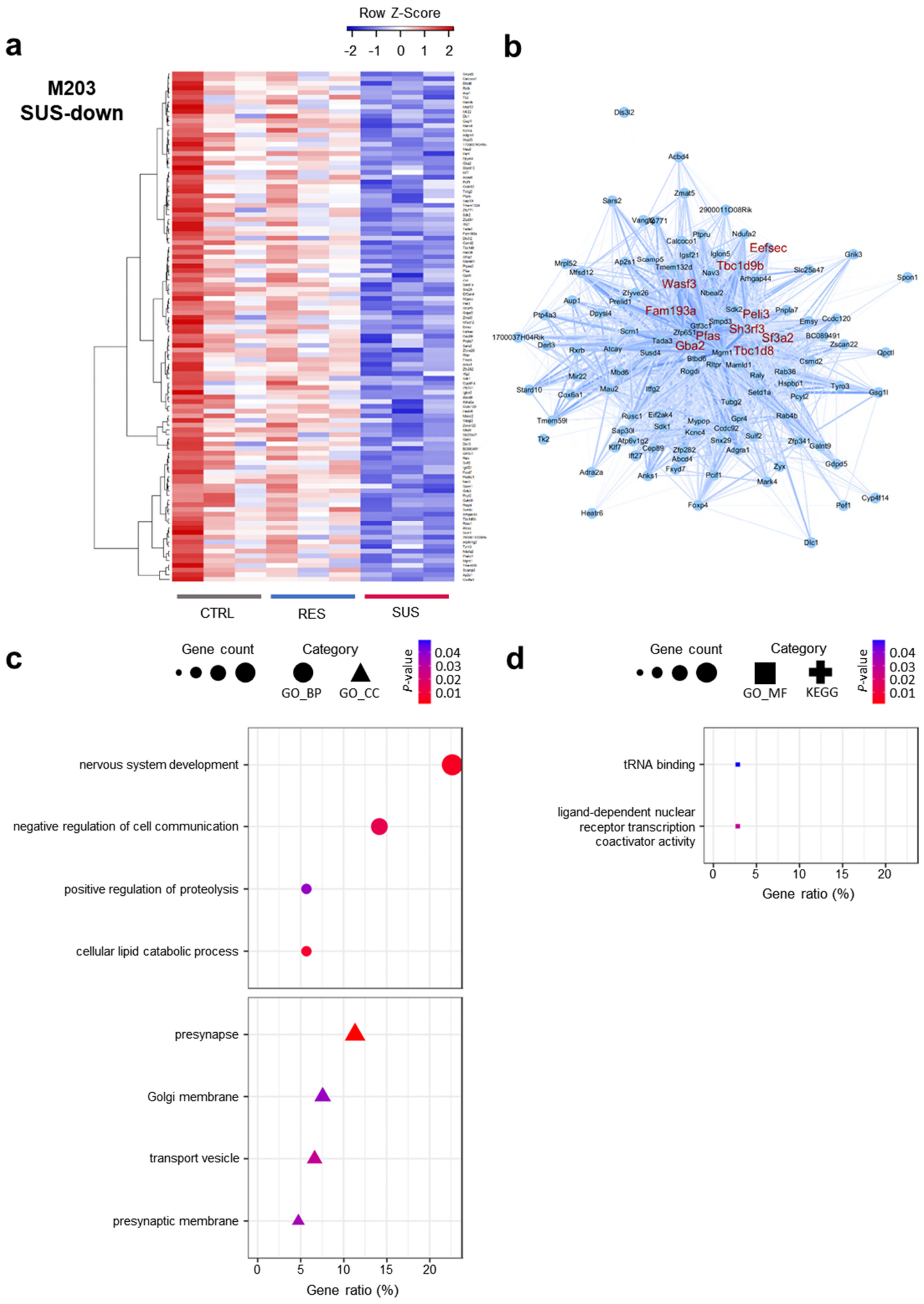
a
M203
SUS-down
Row Z-Score
-2 -1 0 1 2
CTRL
RES
SUS
b
Eefsec
Tbc1d9b
Wasf3
Fam193a
Peli3
Sh3rf3
Pfas
Sf3a2
Gba2
Tbc1d8
c
Gene count
Category
GO_BP
GO_CC
P-value
0.04
0.03
0.02
0.01
nervous system development
negative regulation of cell communication
positive regulation of proteolysis
cellular lipid catabolic process
presynapse
Golgi membrane
transport vesicle
presynaptic membrane
0 5 10 15 20
Gene ratio (%)
d
Gene count
Category
GO_MF
KEGG
P-value
0.04
0.03
0.02
0.01
tRNA binding
ligand-dependent nuclear receptor transcription coactivator activity
0 5 10 15 20
Gene ratio (%)

Supplementary Fig. 11. Characterization of the SUS-down module (M203). (a) Module genes were specifically downregulated in the SUS group. (b) Visualization of the intramodular co-expression network of a gene module specifically repressed in the SUS group. (c) GO enrichment analysis of biological processes and cellular components of the SUS-down module. (d) Enrichment analysis of GO molecular function and KEGG analysis of SUS-down module genes.

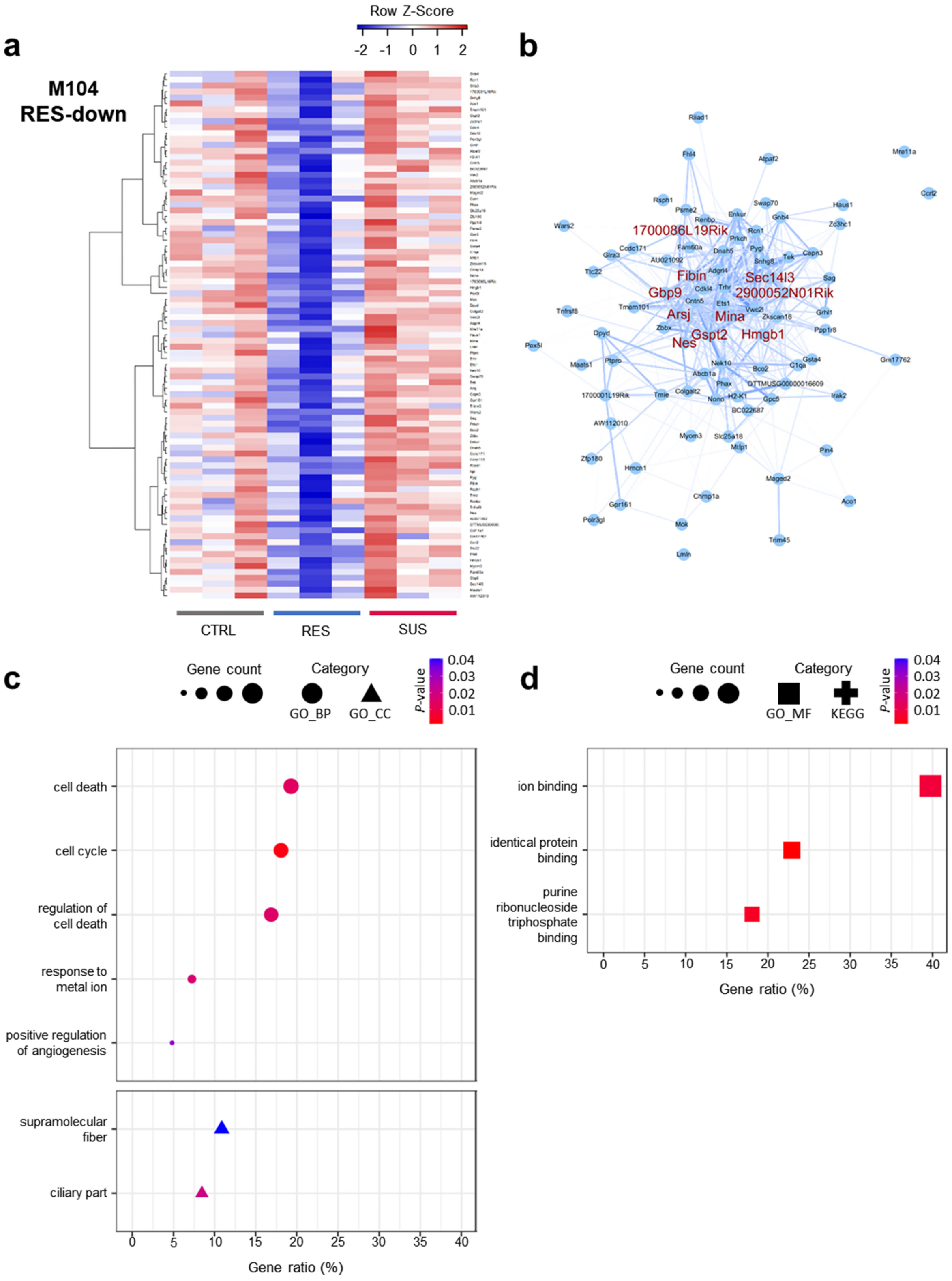

a
b
Row Z-Score
-2 -1 0 1 2
M104
RES-down
CTRL
RES
SUS
1700086L19Rik
Fibin
Sec14l3
Gbp9
2900052N01Rik
Arsj
Mina
Nes
Gspt2
Hmgb1
c
Gene count
Category
GO_BP
GO_CC
p-value
0.04
0.03
0.02
0.01
cell death
cell cycle
regulation of cell death
response to metal ion
positive regulation of angiogenesis
supramolecular fiber
ciliary part
0 5 10 15 20 25 30 35 40
Gene ratio (%)
d
Gene count
Category
GO_MF
KEGG
p-value
0.04
0.03
0.02
0.01
ion binding
identical protein binding
purine ribonucleoside triphosphate binding
0 5 10 15 20 25 30 35 40
Gene ratio (%)

Supplementary Fig. 12. Characterization of the RES-down module (M104). (a) Module genes were specifically downregulated in the RES group. (b) Visualization of the intramodular co-expression network of a gene module specifically repressed in the RES group. (c) GO enrichment analysis of the biological processes and cellular components of the RES-down module. (d) Enrichment analysis of GO molecular function and KEGG analysis of RES-down module genes.

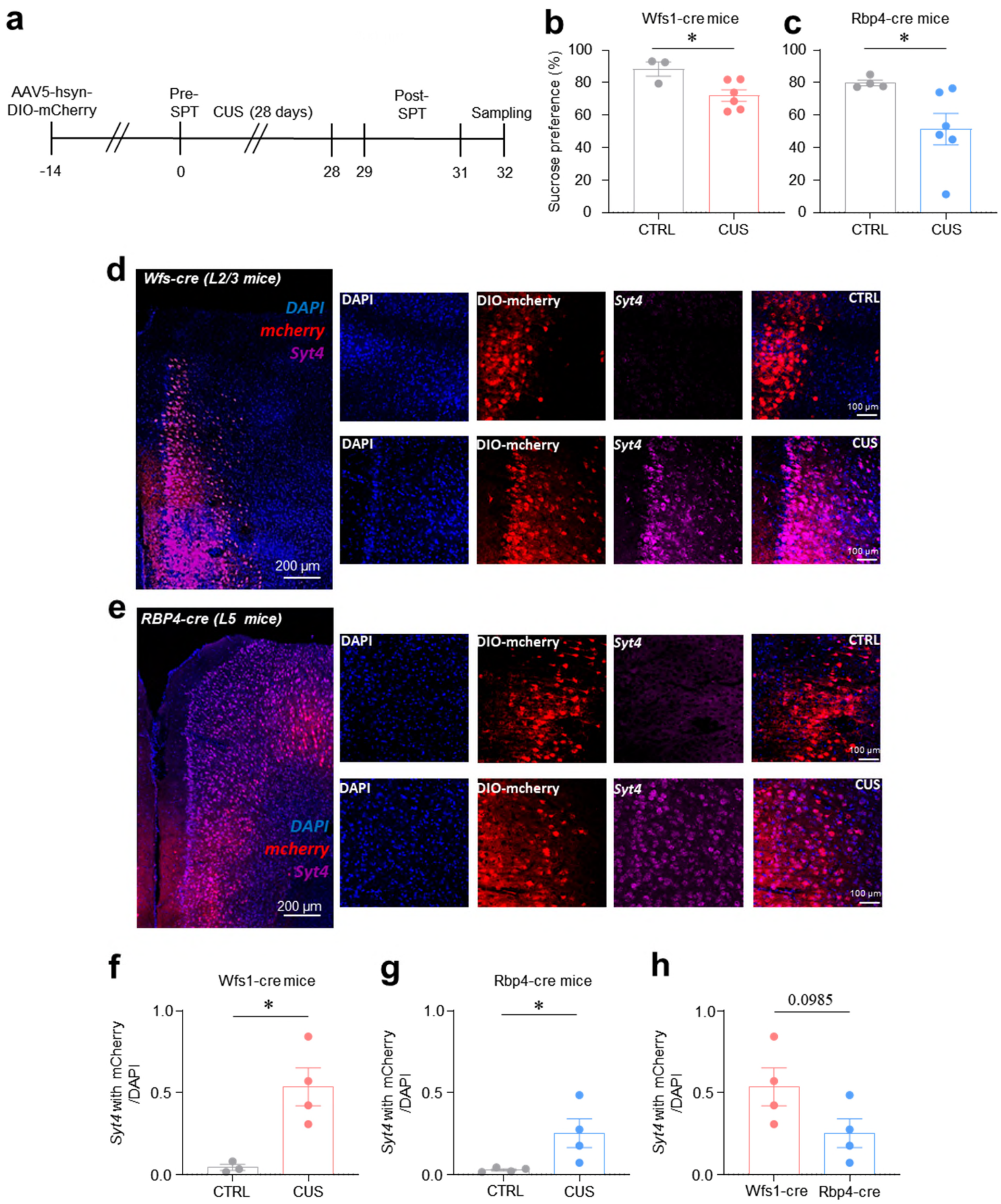
a
AAV5-hsyn-DIO-mCherry
Pre-SPT
CUS (28 days)
Post-SPT
Sampling
-14
0
28
29
31
32
b
Wfs1-cre mice
c
Rbp4-cre mice
Sucrose preference (%)
CTRL
CUS
d
Wfs-cre (L2/3 mice)
DAPI
mcherry
Syt4
DIO-mcherry
200 μm
100 μm
e
RBP4-cre (L5 mice)
f
Wfs1-cre mice
Syt4 with mCherry /DAPI
g
Rbp4-cre mice
h
0.0985
Wfs1-cre
Rbp4-cre

Supplementary Fig. 13. *Syt4* demonstrates widespread expression within the PFC. (a) Experimental procedures for CUS using layer-specific mice. (b–c) Sucrose preference in both *Wfs1*-Cre and *Rbp4*-Cre mice after CUS (b, unpaired t-test, $t_7 = 2.724$, $p = 0.0296$, $n = 3, 6$; c, unpaired t-test, $t_8 = 2.342$, $p = 0.0472$, $n = 4, 6$). **(d–e)** Representative images of fluorescence in situ hybridization for *Syt4* localization in layer-specific mice. (f) Layer 2/3-specific *Syt4* expression in *Wfs1*-Cre mice after CUS (unpaired t-test, $t_5 = 3.579$, $p = 0.0159$, $n = 3, 4$). (g) Layer 5-specific *Syt4* expression in *Rbp4*-Cre mice after CUS (unpaired t-test, $t_6 = 2.540$, $p = 0.0441$, $n = 4$). (h) L2/3 and L5 showed no significant difference in *Syt4* expression after CUS (unpaired t-test, $t_6 = 1.954$, $p = 0.0985$, $n = 4$). $^{*}p < 0.05$, $^{**}p < 0.01$, $^{***}p < 0.001$. The bar graphs show the mean ± SEM.

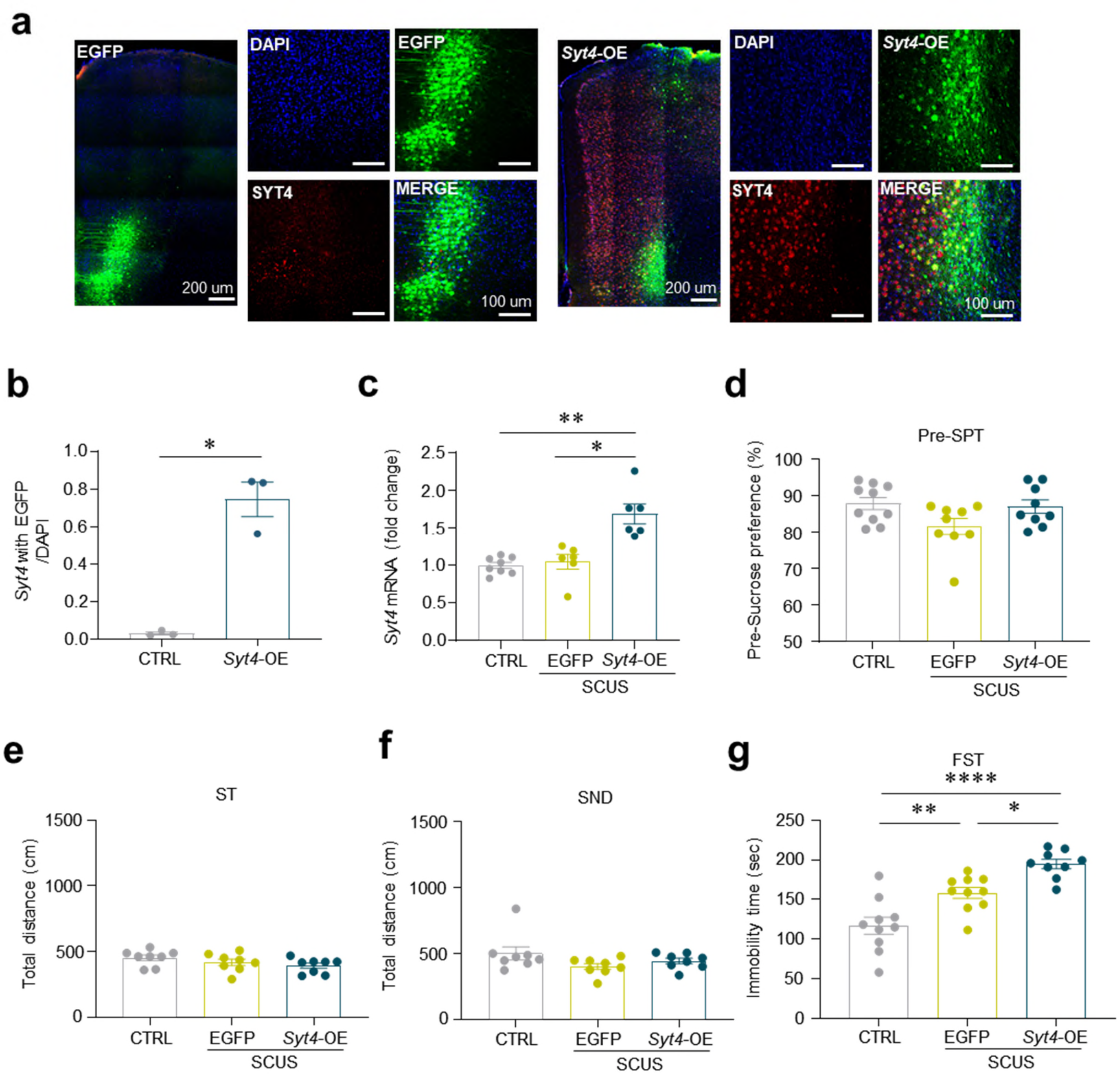
a
EGFP
DAPI
EGFP
SYT4
MERGE
200 um
100 um
Syt4-OE
DAPI
Syt4-OE
SYT4
MERGE
200 um
100 um
b
Syt4 with EGFP /DAPI
CTRL
Syt4-OE
c
Syt4 mRNA (fold change)
CTRL
EGFP
Syt4-OE
SCUS
d
Pre-SPT
Pre-Sucrose preference (%)
CTRL
EGFP
Syt4-OE
SCUS
e
ST
Total distance (cm)
CTRL
EGFP
Syt4-OE
SCUS
f
SND
Total distance (cm)
CTRL
EGFP
Syt4-OE
SCUS
g
FST
Immobility time (sec)
CTRL
EGFP
Syt4-OE
SCUS

Supplementary Fig. 14. *Syt4* overexpression in the mPFC plays a critical role in despair- and anxiety-like behaviors. (a) Validation of AAV5-hSyn1-*Syt4*-EGFP virus. (b–c) Measurement of *Syt4* gene expression in each group (b, unpaired t-test, $t_4 = 7.754$, $p = 0.0015$, $n = 3, 4$; c, one-way ANOVA with Welch's test, $W_{2.000,\ 8.727} = 11.30$, $p = 0.0038$, $n = 6$). (d) The sucrose preference did not differ between groups before receiving CUS (Kruskal–Wallis test with Dunn's multiple comparisons post hoc test, $H = 0.2215$, $p = 0.8952$, $n = 10, 9, 9$). (e) Total distance during ST (Kruskal–Wallis test with Dunn's multiple comparisons post hoc test, $H = 2.459$, $p = 0.2924$, $n = 8, 8, 7$). (f) Total distance travelled during the SND test (Kruskal–Wallis test with Dunn's multiple comparisons post hoc test, $H = 4.317$, $p = 0.1155$, $n = 8, 8, 7$). (g) The *Syt4*-deficient mice (*Syt4*-KD) restored the despair-like behavior caused by CUS (one-way ANOVA with Tukey's multiple comparisons post hoc test, $F_{2,\ 23} = 12.44$, $p = 0.0002$, $n = 7, 9, 10$) $^{*}p < 0.05$, $^{**}p < 0.01$, $^{***}p < 0.001$, $^{****}p < 0.0001$. The bar graphs show the mean ± SEM.

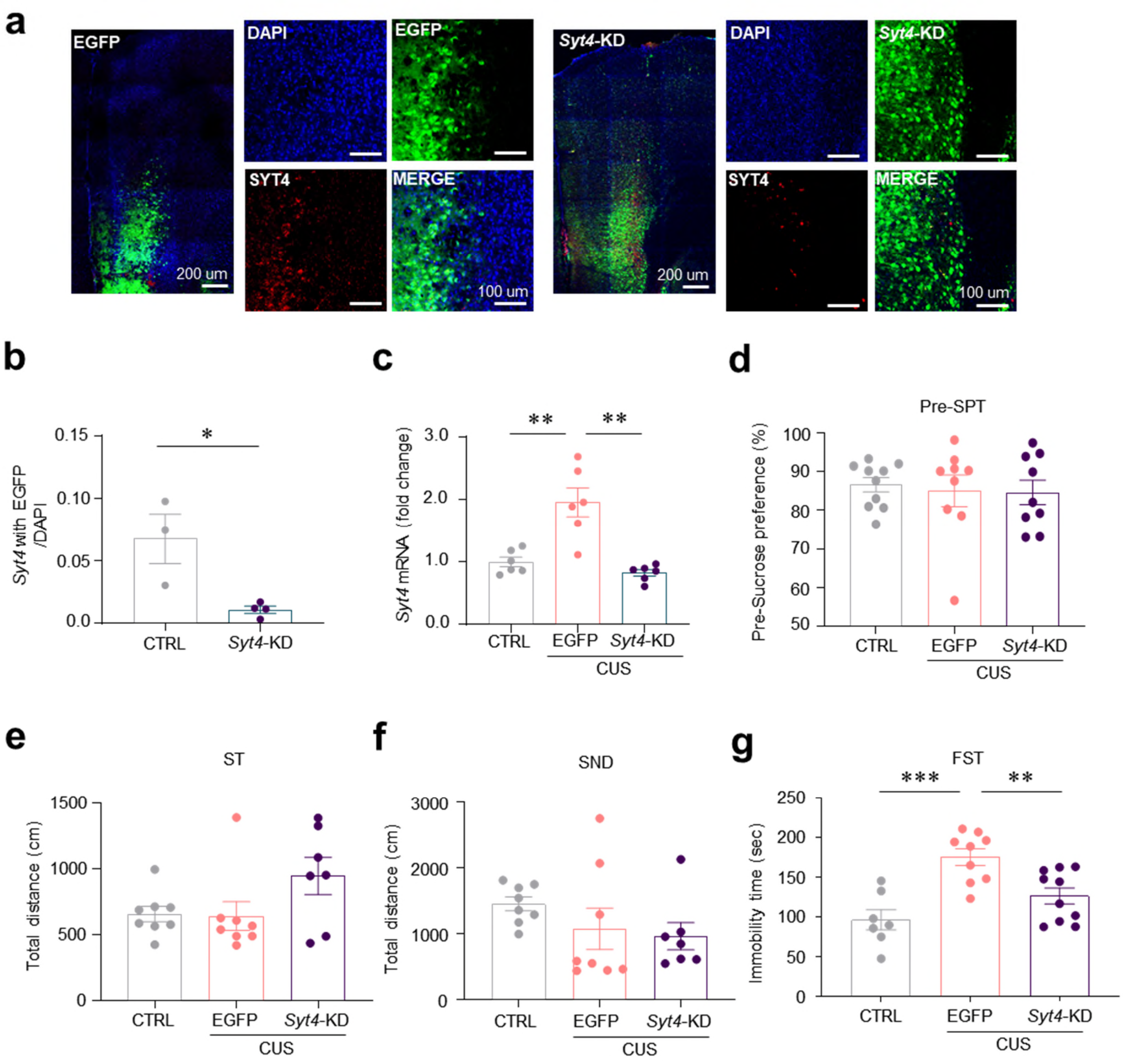

a
EGFP
DAPI
EGFP
SYT4
MERGE
200 um
100 um
Syt4-KD
DAPI
Syt4-KD
SYT4
MERGE
200 um
100 um
b
Syt4 with EGFP /DAPI
0.15
0.10
0.05
0.0
*
CTRL
Syt4-KD
c
Syt4 mRNA (fold change)
3.0
2.0
1.0
0.0
**
**
CTRL
EGFP
Syt4-KD
CUS
d
Pre-SPT
Pre-Sucrose preference (%)
100
90
80
70
60
50
CTRL
EGFP
Syt4-KD
CUS
e
ST
Total distance (cm)
1500
1000
500
0
CTRL
EGFP
Syt4-KD
CUS
f
SND
Total distance (cm)
3000
2000
1000
0
CTRL
EGFP
Syt4-KD
CUS
g
FST
Immobility time (sec)
250
200
150
100
50
0
***
**
CTRL
EGFP
Syt4-KD
CUS

Supplementary Fig. 15. *Syt4* knockdown in the mPFC rescues despair- and anxiety-like behaviors. (a) Validation of the AAV5-hSyn1-*Syt4*-shRNA-EGFP virus. (b–c) Measurement of *Syt4* gene expression in each group (b, unpaired t-test, $t_5 = 3.354$, $p = 0.0202$, $n = 3, 4$; c, Kruskal–Wallis test with Dunn's multiple comparisons post hoc test, $H = 12.68$, $p = 0.0002$, $n = 8, 6, 6$). (d) Sucrose preference test before receiving CUS (Kruskal–Wallis test with Dunn's multiple comparisons post hoc test, $H = 3.808$, $p = 0.1490$, $n = 10, 9, 9$). (e) Total distance during ST (one-way ANOVA with Tukey's multiple comparisons post hoc test, $F_{2, 21} = 1.922$, $p = 0.1712$, $n = 8$). (f) Total distance travelled during the SND test (Kruskal–Wallis test with Dunn's multiple comparisons post hoc test, $H = 3.780$, $p = 0.1511$, $n = 8$). (g) Mice with *Syt4* overexpression showed a significantly increased duration of despair behavior compared to that exhibited by other groups (one-way ANOVA with Tukey's multiple comparisons post hoc test, $F_{2, 26} = 21.93$, $p < 0.0001$, $n = 10, 10, 9$). $^{*}p < 0.05$, $^{**}p < 0.01$, $^{***}p < 0.001$, $^{****}p < 0.0001$. The bar graphs show the mean ± SEM.

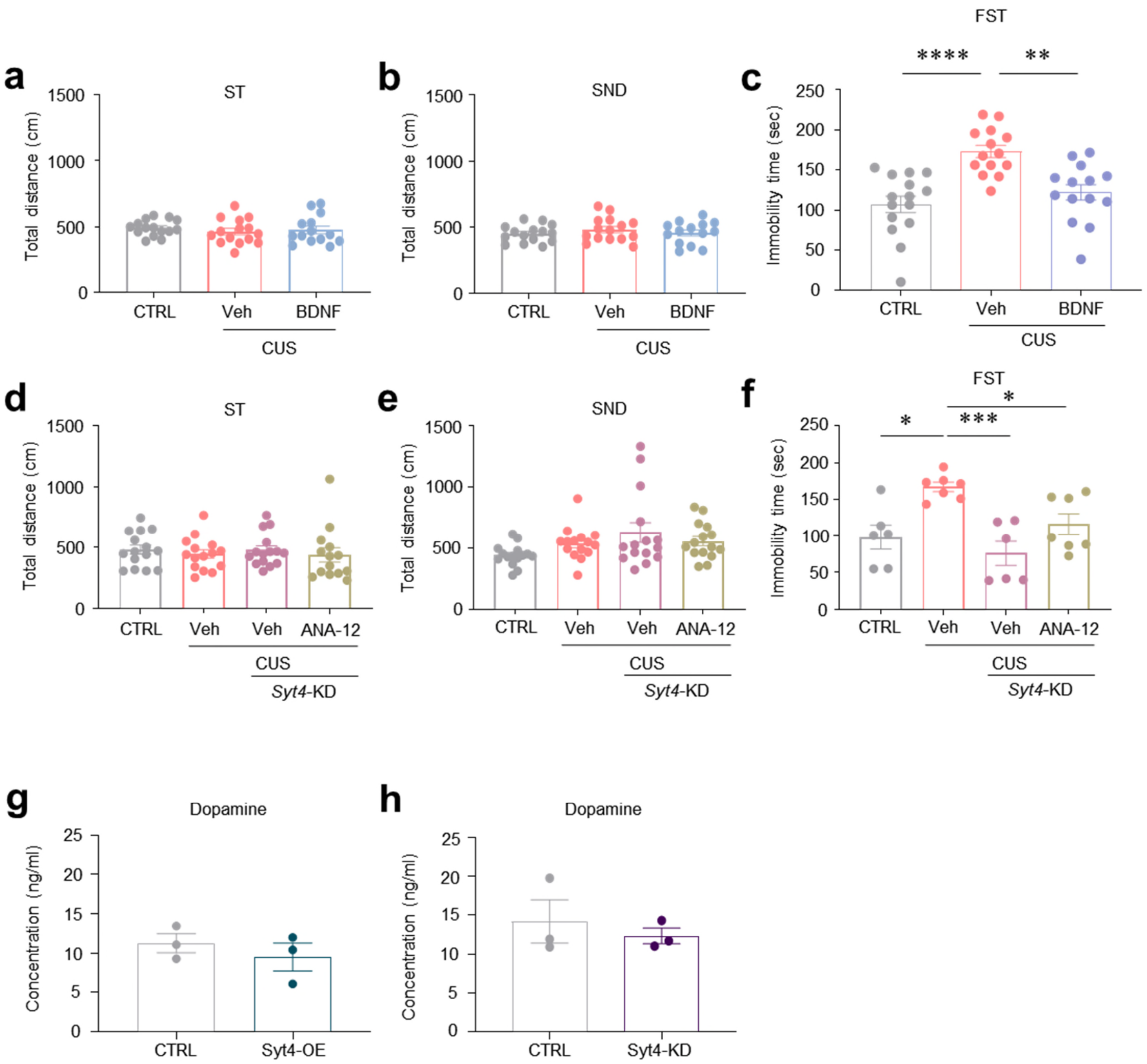
a
ST
Total distance (cm)
1500
1000
500
0
CTRL
Veh
BDNF
CUS
b
SND
Total distance (cm)
CTRL
Veh
BDNF
CUS
c
FST
****
**
Immobility time (sec)
250
200
150
100
50
0
CTRL
Veh
BDNF
CUS
d
ST
Total distance (cm)
CTRL
Veh
Veh
ANA-12
CUS
Syt4-KD
e
SND
Total distance (cm)
CTRL
Veh
Veh
ANA-12
CUS
Syt4-KD
f
FST
*
*
***
Immobility time (sec)
CTRL
Veh
Veh
ANA-12
CUS
Syt4-KD
g
Dopamine
Concentration (ng/ml)
25
20
15
10
5
0
CTRL
Syt4-OE
h
Dopamine
Concentration (ng/ml)
CTRL
Syt4-KD

Supplementary Fig. 16. Regulation of BDNF protein expression by SYT4 in the mPFC and behavioral effects of BDNF on CUS-induced despair and anxiety behaviors. (a) Total distance travelled during the three-chamber sociability test (one-way ANOVA with Tukey's multiple comparisons post hoc test, $F_{2, 41} = 0.3997$, $p = 0.6731$, $n = 15, 15, 14$). (b) Total distance travelled during the three-chamber social novelty test (one-way ANOVA with Tukey's multiple comparisons post hoc test, $F_{2, 41} = 0.6247$, $p = 0.5405$, $n = 15, 15, 14$). (c) Intra-mPFC infusion of BDNF prevented the despair-like behavior induced by CUS in the FST (one-way ANOVA with Tukey's multiple comparisons post hoc test, $F_{2, 40} = 13.58$, $p < 0.0001$, n =15, 14, 14). (d) Total distance travelled during the three-chamber sociability test (Kruskal–Wallis test with Dunn's multiple comparisons post hoc test, $H = 2.896$, $p = 0.4080$, n = 15, 15, 15, 14). (e) Total distance travelled during the three-chamber social novelty test (Kruskal–Wallis test with Dunn's multiple comparisons post hoc test, $H = 8.316$, $p = 0.0399$, n = 15, 15, 15, 15). (f) The *Syt4* deficient mice (*Syt4*-KD) showed restoration of the despair-like behavior caused by CUS. In mice in which Trk-B was suppressed simultaneously, despair behavior increased slightly but not significantly (Kruskal–Wallis test with Dunn's multiple comparisons post hoc test, $H = 12.33$, $p = 0.0063$, $n = 6, 7, 6, 7$). (g) The dopamine concentration was not significantly different between the *Syt4*-EGFP and vehicle control groups (unpaired t-test, t = 0.8274, df = 4, $p = 0.4545$, $n = 3, 3$). (h) The dopamine concentration was not significantly different between the *Syt4*-shRNA and vehicle control groups (unpaired t-test, t = 0.6232, df = 4, $p = 0.5669$, n = 3, 3). $^{*}p < 0.05$, $^{**}p < 0.01$, $^{***}p < 0.001$, $^{****}p < 0.0001$. The bar graphs show the mean ± SEM.

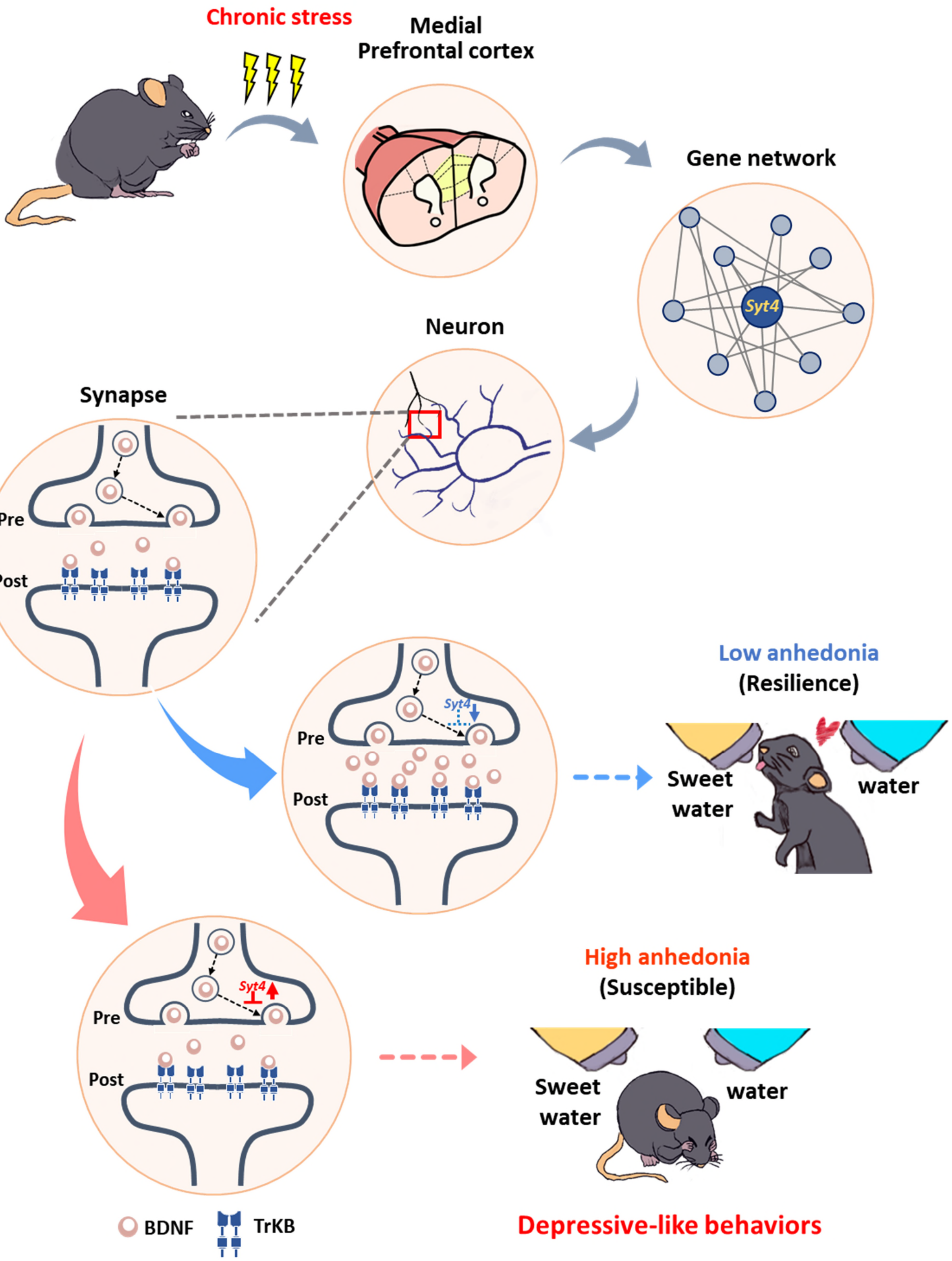
Chronic stress
Medial
Prefrontal cortex
Gene network
Syt4
Neuron
Synapse
Pre
Post
Syt4
Pre
Post
Low anhedonia
(Resilience)
Sweet
water
water
Syt4
Pre
Post
High anhedonia
(Susceptible)
Sweet
water
water
BDNF
TrKB
Depressive-like behaviors

Supplementary Fig. 17. SYT4–BDNF–TrkB system in the mPFC mediates chronic stress-induced anhedonia. Model showing the proposed mechanisms underlying CUS-elicited anhedonic behaviors, which may be mediated by the SYT4–BDNF–TrkB signaling pathway in the mPFC.

Supplementary Tables

Stressor	Duration	Day
Restraint	1 h	1, 6, 10, 15, 18
Light on	Overnight	2, 7, 9, 16, 24
Cage tilt (45°)	Overnight	1, 11, 15, 20, 26
Cage rotation (overcrowding)	1 h	2, 9, 14, 22, 28
Wet bedding (overcrowding)	Overnight	5, 12, 14, 18, 22
Light off	3 h	4, 7, 11, 14, 16, 17, 21, 22, 24
Strobe	Overnight	6, 10, 17, 21, 23, 28
Swim stress (22℃)	5 min	3, 5, 13, 16, 20, 25
Food deprivation	Overnight	3, 8, 13, 19, 25
Overcrowding	Overnight	4, 9,18, 22
Different partner	3 h	8, 12, 19, 23, 26
Water deprivation	Overnight	8, 13, 17, 25

Supplementary Table 1. CUS Experiment Schedule. Common well-validated and authorized stressors were chosen randomly from the following list to ensure that the next stressors remained unpredictable to the subjects. For SCUS, all mice were subjected to two or three daily stimuli for 1 week.

Quality metrics of RNA-sequencing reads

Sample ID	% of processed reads	% of mapped reads (%)	% of unmapped reads (%)
CTRL_1	80,030,862	76,298,704 (95.34%)	3,732,158 (4.66%)
CTRL_2	87,213,872	85,217,805 (97.71%)	1,996,067 (2.29%)
CTRL_3	95,179,400	92,581,914 (97.27%)	2,597,486 (2.73%)
RES_1	74,714,856	66,085,635 (88.45%)	8,629,221 (11.55 %)
RES_2	71,660,514	65,943,942 (92.02%)	5,716,572 (7.98%)
RES_3	59,440,326	54,160,586 (91.12%)	5,279,740 (8.88%)
SUS_1	81,846,116	75,249,430 (91.94%)	6,596,686 (8.06%)
SUS_2	74,126,316	68,750,866 (92.75%)	5,375,450 (7.25 %)
SUS_3	73,433,024	67,903,437 (92.47%)	5,529,587 (7.53%)

Reproducibility between Samples

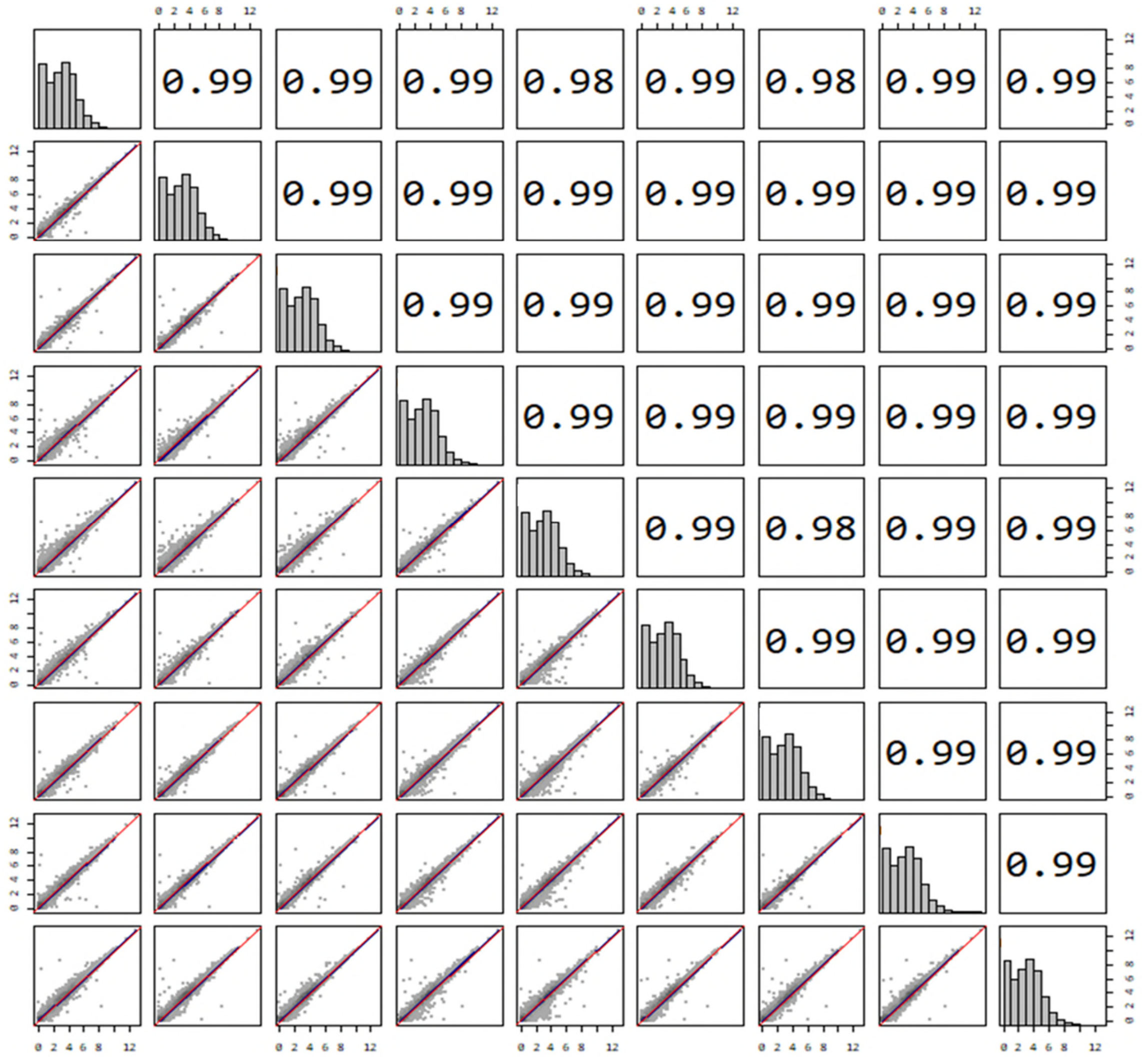

Supplementary Table 2. Quality metrics of RNA sequencing reads and reproducibility between samples. The reproducibility of repeated samples can be confirmed by determining the degree of similarity (Pearson's coefficient) between samples using normalized values for each sample (range: $-1 \leq r \leq 1$). The closer the correlation coefficient value is to 1, the higher the similarity between samples.

Supplementary Table 3. List of DEGs. Genes with differential expression in the mPFC between control vs. CUS mice.

Supplementary Table 4. Functional annotations of DEGs. Gene Ontology (GO) and Kyoto Encyclopedia of Genes and Genomes (KEGG) pathway enrichment analyses of the six DEG groups (SUS-up, SUS-down, RES-up, RES-down, RES-up/SUS-down, SUS-up/RES-down) to identify the biological role of DEGs.

Supplementary Table 5. List of module genes. WGCNA was employed to discover behavioral phenotype-specific genes, which led to the identification of 203 co-expressed gene modules.

Supplementary Table 6. Module-trait relationships. Relationships between modules and sample traits through linear regression and Pearson correlation.

Supplementary Table 7. Functional annotations of modules. GO and KEGG pathway enrichment analyses were performed as in the DEG analysis to confirm the biological function of the module.

SI References

1. Koo, J.W., Russo, S.J., Ferguson, D., Nestler, E.J. & Duman, R.S. Nuclear factor-kappaB is a critical mediator of stress-impaired neurogenesis and depressive behavior. *Proc Natl Acad Sci U S A* **107**, 2669-2674 (2010).
2. Monteggia, L.M., *et al.* Brain-derived neurotrophic factor conditional knockouts show gender differences in depression-related behaviors. *Biol Psychiatry* **61**, 187-197 (2007).
3. Rein, B., Ma, K. & Yan, Z. A standardized social preference protocol for measuring social deficits in mouse models of autism. *Nat Protoc* **15**, 3464-3477 (2020).
4. Shirayama, Y., Chen, A.C., Nakagawa, S., Russell, D.S. & Duman, R.S. Brain-derived neurotrophic factor produces antidepressant effects in behavioral models of depression. *J Neurosci* **22**, 3251-3261 (2002).
5. Azogu, I. & Plamondon, H. Inhibition of TrkB at the nucleus accumbens, using ANA-12, regulates basal and stress-induced orexin A expression within the mesolimbic system and affects anxiety, sociability and motivation. *Neuropharmacology* **125**, 129-145 (2017).
6. Kim, J., Kang, S., Choi, T.Y., Chang, K.A. & Koo, J.W. Metabotropic Glutamate Receptor 5 in Amygdala Target Neurons Regulates Susceptibility to Chronic Social Stress. *Biol Psychiatry* **92**, 104-115 (2022).
7. Guenthner, C.J., Miyamichi, K., Yang, H.H., Heller, H.C. & Luo, L. Permanent genetic access to transiently active neurons via TRAP: targeted recombination in active populations. *Neuron* **78**, 773-784 (2013).
8. Anastasia, A., *et al.* Val66Met polymorphism of BDNF alters prodomain structure to induce neuronal growth cone retraction. *Nat Commun* **4**, 2490 (2013).
9. Lim, J.Y., Reighard, C.P. & Crowther, D.C. The pro-domains of neurotrophins, including BDNF, are linked to Alzheimer's disease through a toxic synergy with Abeta. *Hum Mol Genet* **24**, 3929-3938 (2015).
10. Mehravaran, S., Dehzangi, I. & Rahman, M.M. Interocular Symmetry Analysis of Corneal Elevation Using the Fellow Eye as the Reference Surface and Machine Learning. *Healthcare-Basel* **9** (2021).
11. Abdi, H. & Williams, L.J. Principal component analysis. *WIREs Computational Statistics* **2**, 433-459 (2010).
12. Wu, S.J., *et al.* Cortical somatostatin interneuron subtypes form cell-type-specific circuits. *Neuron* **111**, 2675-2692 e2679 (2023).
